# The FSH–mTOR–CNP signaling axis initiates follicular antrum formation by regulating tight junction, ion pumps, and aquaporins

**DOI:** 10.1016/j.jbc.2023.105015

**Published:** 2023-07-05

**Authors:** Xiaodong Wang, Shanshan Zhou, Zian Wu, Ruiyan Liu, Zaohong Ran, Jianning Liao, Hongru Shi, Feng Wang, Jianguo Chen, Guoshi Liu, Aixin Liang, Liguo Yang, Shujun Zhang, Xiang Li, Changjiu He

**Affiliations:** 1 National Center for International Research on Animal Genetics, Breeding and Reproduction/Key Laboratory of Agricultural Animal Genetics, Breeding and Reproduction of Ministry of Education, College of Animal Sciences and Technology/Veterinary Medicine, Huazhong Agricultural University, Wuhan, China; 2 Department of Molecular, Cell and Cancer Biology, University of Massachusetts Chan Medical School, Worcester, USA; 3 Key Laboratory of Animal Genetics and Breeding of the Ministry of Agriculture, College of Animal Science and Technology, China Agricultural University, Beijing, China

**Keywords:** follicle, follicular antrum, FSH, CNP, mTOR, tight junction, ion pump, aquaporin

## Abstract

The initial formation of the follicular antrum (iFFA) serves as a dividing line between gonadotropin-independent and gonadotropin-dependent folliculogenesis, enabling the follicle to sensitively respond to gonadotropins for its further development. However, the mechanism underlying iFFA remains elusive. Herein, we reported that iFFA is characterized by enhanced fluid absorption, energy consumption, secretion, and proliferation and shares a regulatory mechanism with blastula cavity formation. By use of bioinformatics analysis, follicular culture, RNA interference, and other techniques, we further demonstrated that the tight junction, ion pumps, and aquaporins are essential for follicular fluid accumulation during iFFA, as a deficiency of any one of these negatively impacts fluid accumulation and antrum formation. The intraovarian mammalian target of rapamycin–C-type natriuretic peptide pathway, activated by follicle-stimulating hormone, initiated iFFA by activating tight junction, ion pumps, and aquaporins. Building on this, we promoted iFFA by transiently activating mammalian target of rapamycin in cultured follicles and significantly increased oocyte yield. These findings represent a significant advancement in iFFA research, further enhancing our understanding of folliculogenesis in mammals.

As the functional unit in the ovary, the follicle supports the female’s estrous cycle, secondary sexual characteristics, and fertility (1, 2). Folliculogenesis, a process of follicle development, can be divided into two distinct phases, that is, the gonadotropin-independent and the gonadotropin-dependent phases. The gonadotropin-independent phase begins with primordial follicle activation and ends before the initial formation of follicular antrum (iFFA). During this phase, the follicles have low responsiveness to gonadotropins because of the scarcity of gonadotropin receptors (3). On the other hand, the gonadotropin-dependent phase initiates with iFFA and completes with ovulation. At this phase, small antra form within the follicle, and the follicle eventually matures and ruptures under the stimulation of gonadotropins (4). Therefore, iFFA, characterized by both the scattered emergence of small antra and the accumulation of fluid, is considered as a dividing line between gonadotropin-independent and gonadotropin-dependent folliculogenesis. Obviously, iFFA plays a vital role in folliculogenesis as it enables the follicle to respond sensitively to gonadotropins for its further development.

Currently, through the use of gene editing, single-cell genomics, spatial genomics, and cell lineage tracing, the potential mechanisms of folliculogenesis are being progressively unveiled. For example, the key proteins synthesized by oocyte or follicular cells, including GDF9, SOHLH1/2, NOBOX, LHX8, FIGLA, and LFNG, are identified to regulate folliculogenesis (5, 6, 7, 8); mammalian target of rapamycin (mTOR)–Kit–Akt–Foxo3 has been confirmed as a key pathway to control follicle activation (9, 10, 11); the developmental dynamics of folliculogenesis (12, 13, 14, 15), the cytological events, and their underlying regulatory signaling during gonadotropin-dependent folliculogenesis have been extensively investigated (16, 17). However, the studies on iFFA are lagged behind. For example, the latest review on iFFA was published in 2010 (18), and only handful research articles on iFFA could be retrieved from the PubMed database (19, 20, 21, 22, 23, 24, 25, 26, 27, 28, 29, 30, 31, 32, 33, 34, 35, 36, 37, 38, 39, 40, 41, 42). In addition, the research targets are relative narrow. These include that cytokines (GDF9, BMP15, Kit ligand, and epidermal growth factor) secreted by the oocyte and follicular cell affect iFFA in the cultured follicles (19, 20, 21, 22, 23, 24); the osmotic pressure gradient created by mucopolysaccharides and proteoglycans is believed to be the power source of fluid for entry into the follicular antrum (25); follicle-stimulating hormone receptor (*FSHR*)-, *FSHβ*-, and *IGF1*-deficient mice have iFFA failure (26, 27, 28, 29), whereas *ESR1-* and *CYP19A1*-deficient mice have abnormally large follicular antra (30, 31); aquaporins are associated with the formation of follicular antrum, but inexplicably, aquaporin-8 deficiency led to increased size of follicular antrum either *in vivo* or *in vitro* (32, 33). Overall, iFFA remains a mysterious aspect of folliculogenesis, despite its physiological importance. Two potential reasons may hind the iFFA's research. One is that unlike follicular antrum enlargement, iFFA is a subtle process that lacks any notable morphological or endocrine-induced behavioral changes (43). The other is that determination of the exact timing of iFFA research is challenging because it occurs asynchronously during the continuous process of folliculogenesis without a fixed time frame.

In recent years, the unique developmental dynamics of mouse folliculogenesis have been uncovered, creating an opportunity to determine the ideal timing for researching iFFA. Specifically, a cohort of follicles are preassembled in the ovarian medulla during the fetal period. Once formed, these follicles are synchronously activated and become the first wave of folliculogenesis (12, 13, 14). In the current study, the time frame for researching iFFA was obtained by tracking the first wave of folliculogenesis in juvenile mice. Within this time frame, RNA-Seq and experiments were conducted to investigate the changes in follicular cells' life activities during iFFA, the signaling pathway regulating iFFA, and the mechanism underlying follicular fluid production. These findings are crucial for comprehending the process of establishing the follicular antrum from scratch.

## Results

### Postnatal day 14 to 16 was the time frame for studying iFFA *in vivo*

**Figure 1:**
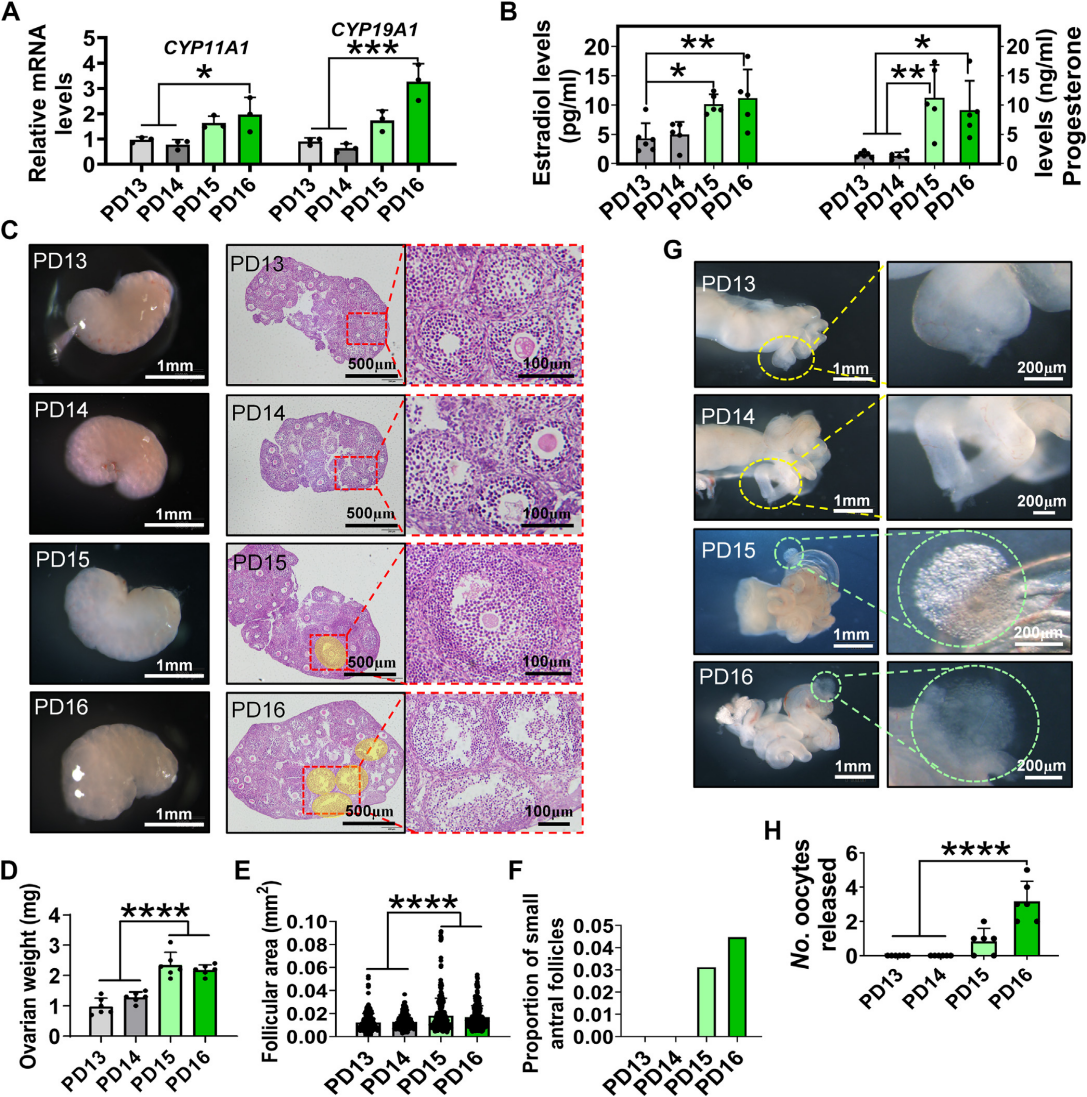
**PD14–16 was the time frame for studying iFFA *in vivo*.***A*, expression of genes indicating follicular cell differentiation, n = 3 ovaries, collected from three mice. *B*, serum levels of estradiol and progesterone, n = 6 (PD13) and 5 (PD14–16) mice. *C*, representative photographs of ovaries and ovarian slices, the areas covered in *yellow* are small antral follicles. *D*, ovarian weight, n = 6 biologically independent ovaries. *E*, the follicular area. n = 301 (PD13), 386 (PD14), 425 (PD15), and 364 (PD16) follicles, from three different ovaries. *F*, proportion of small antral follicles. *G*, representative photographs showed ovaries responding to superovulation as early as PD15. The oocytes are surrounded by *green circles*. *H*, statistical chart of the oocytes released, n = 6 oviducts, collected from three mice. Statistical significance was determined using one-way ANOVA followed by Tukey’s post hoc test. Values are mean ± SD. ∗*p* < 0.05, ∗∗*p* < 0.01, ∗∗∗*p* < 0.001, and ∗∗∗∗*p* < 0.0001. The experiments of *A*, *B*, *D*, and *H* were repeated three times, and similar results were obtained. iFFA, initial formation of the follicular antrum; PD, postnatal day.

To determine the time frame for iFFA, juvenile mouse ovaries from postnatal day (PD) 13 to 16 were isolated to examine the indicators related to follicular cell differentiation since iFFA is accompanied by the differentiation of follicular cells (23). Quantitative RT–PCR (qRT–PCR) analysis showed that the expression of *CYP11A1* and *CYP19A1*, which indicate follicular cell differentiation, increased on PD15 and 16 (Fig. 1*A*). In correlation with gene expression, the serum levels of estradiol and progesterone increased on PD15 and 16 (Fig. 1*B*). The morphological analysis from the collected ovaries also showed a sudden increase in ovarian weight (Fig. 1, *C* and *D*) and follicular area (Fig. 1, *C* and *E*) on PD15 and 16. Moreover, the earliest appearance of follicular antra was observed in the ovaries of PD15 (Fig. 1, *C* and *F*). Since only small antral follicles can respond to gonadotropin in prepubertal ovaries, we conducted superovulation to verify the emergence time of iFFA. Consistent with morphological changes, the release of oocytes in the oviducts of mice on PD15 and 16 after superovulation treatment was observed (Fig. 1, *G* and *H*). These data supported that PD14 to 16 is the designated time frame for studying mouse iFFA *in vivo*.

### The alterations of cellular events occurring during iFFA uncovered by bioinformatics analysis

**Figure 2:**
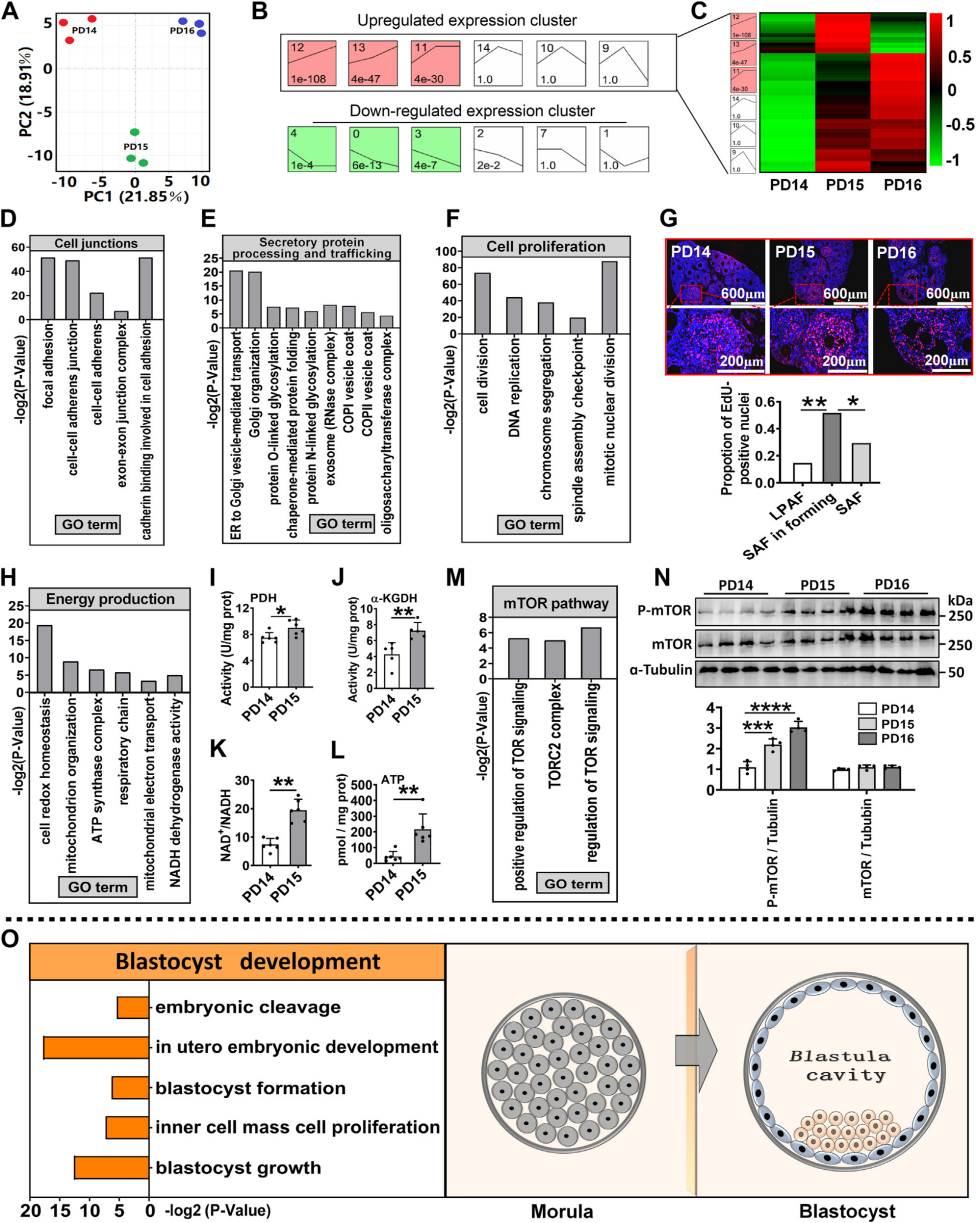
**The alterations of cellular events occurring during iFFA uncovered by bioinformatics analysis.***A*, principal component analysis of the change in transcriptome, n = 3 biologically independent ovaries. *B*, classification of gene expression clusters. *C*, heat map of genes in upregulated expression cluster. *D*, cytological events related to “cell junctions.” *E*, cytological events related to “secretory protein processing and trafficking.” *F*, cytological events related to “cell proliferation.” *G*, follicular cell proliferation measured by EdU *in vivo* staining. The red dots represented EdU-positive nuclei, n = 3 biologically independent sections, the experiments were repeated two times independently, and similar results were obtained. *H*, cytological events related to “energy production.” *I*, activity of pyruvate dehydrogenase (PDH), n = 6 biologically independent ovaries. *J*, activity of ɑ-ketoglutaricdehydrogenase (ɑ-KGDH), n = 5 biologically independent ovaries. *K*, ratio of NAD^+^/NADH, n = 6 biologically independent ovaries. *L*, ATP contents, n = 6 biologically independent ovaries. *M*, cytological events related to “mTOR pathway.” *N*, phosphorylation of mTOR in ovaries. The original blots can be viewed in Fig. S16. *O*, cytological events related to “blastocyst development.” *Left*: GO analysis, *right*: schematic diagram of blastula cavity formation. Statistical significance was determined using two-tailed unpaired Student’s *t* test or Chi-squared test (Fig. 2*G*). Values are mean ± SD. ∗*p* < 0.05, ∗∗*p* < 0.01, ∗∗∗*p* < 0.001, and ∗∗∗∗*p* < 0.0001. The experiments of *I*, *J*, *K*, *L*, and *N* were repeated three times, and similar results were obtained. GO, Gene Ontology; iFFA, initial formation of the follicular antrum; LPAF, large preantral follicle; mTOR, mammalian target of rapamycin; SAF, small antral follicle.

To investigate the regulatory mechanism underlying iFFA, the cellular activities within ovaries from PD14 to 16 were analyzed by RNA-Seq. The principal component analysis revealed significant differences in transcriptional profile during iFFA (Fig. 2*A*). The Short Time-series Expression Miner (STEM) analysis identified two distinguishable gene expression clusters during iFFA, that is, the upregulated and the downregulated expression clusters (Fig. 2*B*).

Gene Ontology (GO) analysis of the genes in upregulated expression cluster (Fig. 2*C*) has revealed activations of multiple cytological events during iFFA. These events are associated with “transcription” (Fig. S1*A*), “epigenetic modification” (Fig. S1*B*), and “cell junctions” (Fig. 2*D*). Cytological events related to “secretory/membrane protein processing and trafficking” have also been identified (Fig. 2*E*). Kyoto Encyclopedia of Genes and Genomes analysis suggested that the upregulated genes encoding secretory proteins were enriched in *mTOR*, *PI3K-AKT*, *TGF-beta*, and *Wnt* pathways (Fig. S1*C*). In addition, the expression of genes encoding large macromolecule hydrophilic secretory proteins, including *ITIH2*, *ITIH4*, *HAS2*, *CSPG4*, and *TNFATP*, also increased during iFFA (Fig. S2). Previous studies (25) reported that these proteins could increase the osmotic pressure gradient, suggesting that their increase during iFFA may be facilitating fluid absorption. Cytological events related to “cell proliferation” were identified (Fig. 2*F*). EdU staining suggested that cell division was enhanced during the small antral formation but slowed down after antra formation (Fig. 2*G*). Cytological events related to energy production were identified (Fig. 2*H*). Confirmation experiments revealed an upregulation of the glucose transporter gene *SLC2A10* on PD15 (Fig. S3*A*), along with genes encoding rate-limiting enzymes of glycolysis (*PFKM* and *PKM*) and tricarboxylic acid cycle (*PDHA*, *CS*, and *OGDH*) (Fig. S3, *B* and *C*). Consistent with gene expression, the activities of pyruvate dehydrogenase and ɑ-ketoglutaricdehydrogenase in ovarian homogenates were enhanced on PD15 (Fig. 2, *I* and *J*). In addition, NAD^+^/NADH, a reflector of electron transport chain activity, and ATP content in ovarian homogenate increased on PD15, respectively (Fig. 2, *K* and *L*). GO analysis indicated enhanced mTOR signaling pathway during iFFA (Fig. 2*M*), and this was confirmed by Western blot analysis with an increased mTOR phosphorylation (Fig. 2*N*). Collectively, these data demonstrate that iFFA is a process characterized by enhanced fluid absorption, energy consumption, secretion, proliferation, and mTOR activation.

In particular, GO analysis revealed that several biological events related to blastocyst development were also identified (*e.g.*, in utero embryonic development, blastocyst formation, inner cell mass cell proliferation, and blastocyst growth, etc.). This implies that iFFA may share a regulatory mechanism with blastula cavity formation. From a morphological standpoint, the formation of follicular antrum and blastula cavity are two highly comparable processes (Fig. 2*O*).

### Tight junction, ion pumps, and aquaporins were essential for follicular fluid accumulation during iFFA

**Figure 3:**
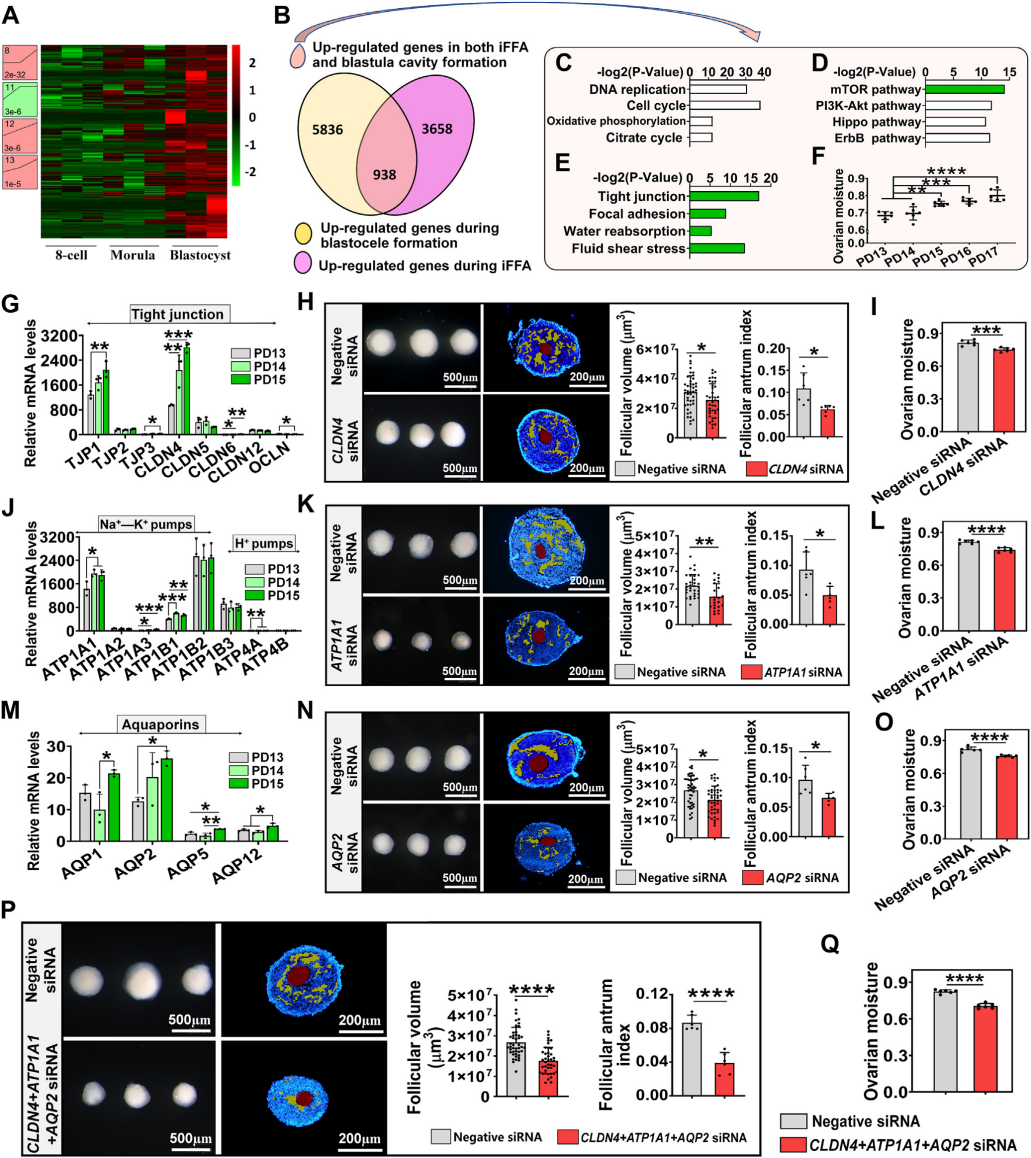
**Tight junction, ion pumps, and aquaporins were essential for follicular fluid accumulation during iFFA.***A*–*F*, common regulatory factors of iFFA and blastula cavity formation analyzed by bioinformatics. *A*, heat map of the transcriptome during blastula cavity formation (based on reanalysis of previously published transcriptome data). *B*, venn diagram shown the overlapping differentially expressed genes between iFFA and blastula cavity formation. *C*, GO analysis showed that cell proliferation and energy production increase during iFFA and blastula cavity formation. *D*, GO analysis showed that mTOR activates during iFFA and blastula cavity formation. *E*, GO analysis showed that fluid absorption increases during iFFA and blastula cavity formation. *F*, change in ovarian moisture during iFFA, n = 6 biologically independent ovaries. *G*, genes encoding the core components of tight junction, n = 3 biologically independent ovaries. *H*, changes in the follicular volume (n = negative siRNA: 45 follicles; *CLDN4* siRNA: 40 follicles) and follicular antrum index (n = 6 follicles) after *CLDN4* knockdown. The scrambled shRNA was used as negative siRNA control. The areas covered in *yellow* were the scattered follicular antra, and the areas covered in *red* were oocytes. *I*, changes in the ovarian moisture after *CLDN4* knockdown, n = 6 ovaries, collected from three mice. *J*, changes in the expression of genes encoding ion pumps, n = 3 biologically independent ovaries. *K*, changes in the follicular volume (n = negative siRNA: 32 follicles; *ATP1A1* siRNA: 23 follicles) and follicular antrum index (n = 6 follicles) after *ATP1A1* knockdown. *L*, changes in the ovarian moisture after *ATP1A1* knockdown, n = 6 ovaries, collected from three mice. *M*, changes in the expression of genes encoding aquaporins, n = 3 biologically independent ovaries. *N*, changes in the follicular volume (n = negative siRNA: 46 follicles; *AQP2* siRNA: 45 follicles) and follicular antrum index (n = 6 follicles) after *AQP2* knockdown. *O*, changes in the ovarian moisture after *AQP2* knockdown, n = 6 ovaries, collected from three mice. *P* and *Q*, effects of simultaneous knockdown of *CLDN4*, *ATP1A1*, and *AQP2* on iFFA. *P*, changes in follicular volume (n = negative siRNA: 44 follicles; *CLDN4* + *ATP1A1* + *AQP2* siRNA: 40 follicles) and follicular antrum index (n = 6 follicles). *Q*, changes in the ovarian moisture, n = 6 ovaries, collected from three mice. Statistical significance was determined using one-way ANOVA followed by Tukey’s post hoc test (Fig. 3, *F*, *G*, *J* and *M*) and two-tailed unpaired Student’s *t* test (Fig. 3, *H*, *I*, *K*, *L*, *N*, *O*, *P* and *Q*). Values are mean ± SD. ∗*p* < 0.05, ∗∗*p* < 0.01, ∗∗∗*p* < 0.001, and ∗∗∗∗*p* < 0.0001. The experiments of *F*, *G*, *J*, and *M* were repeated three times, and the experiments of *H*, *I*, *K*, *L*, *N*, *O*, *P*, and *Q* were repeated two times; in both, similar results were obtained. GO, Gene Ontology; iFFA, initial formation of the follicular antrum; mTOR, mammalian target of rapamycin.

The most intriguing information revealed by the GO analysis is that iFFA and blastula cavity formation may share a regulatory mechanism (Fig. 2*O*). This indicates that identifying the regulatory factors they share could potentially expedite the study of iFFA. In light of this, we identified the genes that are upregulated during both iFFA and blastula cavity formation (Fig. 3, *A* and *B*). GO analysis showed that, apart from mTOR signaling, proliferation, and energy production (Fig. 3, *C* and *D*), the genes upregulated during both process were mainly enriched in cytological events associated with fluid accumulation (*e.g.*, tight junction, water reabsorption, fluid shear stress, etc.) (Fig. 3*E*). In line with the GO analysis, there was a significant increase in ovarian moisture on PD15 (Fig. 3*F*), when the follicular antra form. Follicular fluid accumulation is the driving force of iFFA and antrum expansion. From the point of view of biophysics, three prerequisites must be satisfied for fluid to enter and accumulate within the tissue: an osmotic pressure gradient inside and outside the tissue, an enclosed space within the tissue to maintain the osmotic pressure gradient and store ingested fluid, and a rapid water transport channel within the tissue.

We hypothesized that tight junction, a structure that enhances cell–cell adhesion (44), might be the key structure for establishment of the enclosed space within the follicle. This hypothesis was based on the observations of the emergence of tight junction during both iFFA and blastula cavity formation (Figs. 3*E* and S4), and the upregulation of genes encoding the core components of tight junction, including *CLDN4*, which has the highest mRNA abundance (Figs. 3*G* and S5*A*). To validate this speculation, preantral follicles were isolated and transfected with the lentivirus-mediated *CLDN4*-interference plasmids (Fig. S6, *A* and *B*). This process significantly decreased follicular volume and follicular antrum index (Fig. 3*H*) with the downregulation of genes related to follicular cell differentiation including *CYP11A1* and *CYP19A1* (Fig. S6*C*). To further confirm the role of tight junctions in follicular fluid accumulation, the plasmids were directly injected into the ovaries before iFFA (Fig. S7, *A* and *B*). The result showed that ovarian moisture was significantly reduced after *CLDN4* knockdown (Fig. 3*I*). All the results support that tight junction is the key structure for follicular fluid accumulation during iFFA.

Then, the ion pumps were speculated to be the maintainer of osmotic pressure gradient that drive follicular fluid absorption. This was supported by the significant upregulation of genes coding for ion pumps during both iFFA and blastula cavity formation, including *ATP1A1* (encoding Na^+^–K^+^ pump) with the highest mRNA abundance (Figs. 3*J* and S5*B*). In order to test this hypothesis, *ATP1A1* was knocked down (Fig. S6*D*), which significantly reduced follicular volume, follicular antrum index (Fig. 3*K*), and the expression of *CYP19A1* (Fig. S6*E*) in cultured follicles. In the ovary, *ATP1A1* knockdown also resulted in a significant reduction in ovarian moisture (Figs. 3*L* and S7*C*). These data suggest that Na^+^–K^+^ pumps are actively involved in driving follicular fluid absorption during iFFA.

We further hypothesized that the aquaporins may have a role in guiding fluid transport during iFFA since the genes encoding aquaporins were upregulated during both iFFA and blastula cavity formation with the *AQP2* having the highest mRNA abundance (Figs. 3*M* and S5*C*). *AQP2* knockdown in the cultured follicles (Fig. S6*F*) lead to a significant reduction in follicular volume, follicular antrum index (Fig. 3*N*), and the expression of *CYP11A1* (Fig. S6*G*). Similarly, *AQP2* knockdown in the ovary also resulted in a significant reduction in ovarian moisture (Figs. 3*O* and S7*D*). These data suggested that aquaporin-mediated transport plays a key role in facilitating follicular fluid flow into the antra during iFFA.

Finally, *CLDN4*, *ATP1A1*, and *AQP2* were simultaneously knocked down in the cultured follicles and ovaries. Although the interference efficiency of simultaneous knockdown of the three genes was not as good as that of single gene knockdown (Figs. S6*H* and S7*E*), it still resulted in a more significant decrease in the follicular volume, follicular antrum index (Fig. 3*P*), ovarian moisture (Fig. 3*Q*), and the expression of *CYP11A1* and *CYP19A1* (Fig. S6*I*).

### Regulation of C-type natriuretic peptide on iFFA was related to its upregulation of tight junctions, ion pumps, and aquaporins

**Figure 4:**
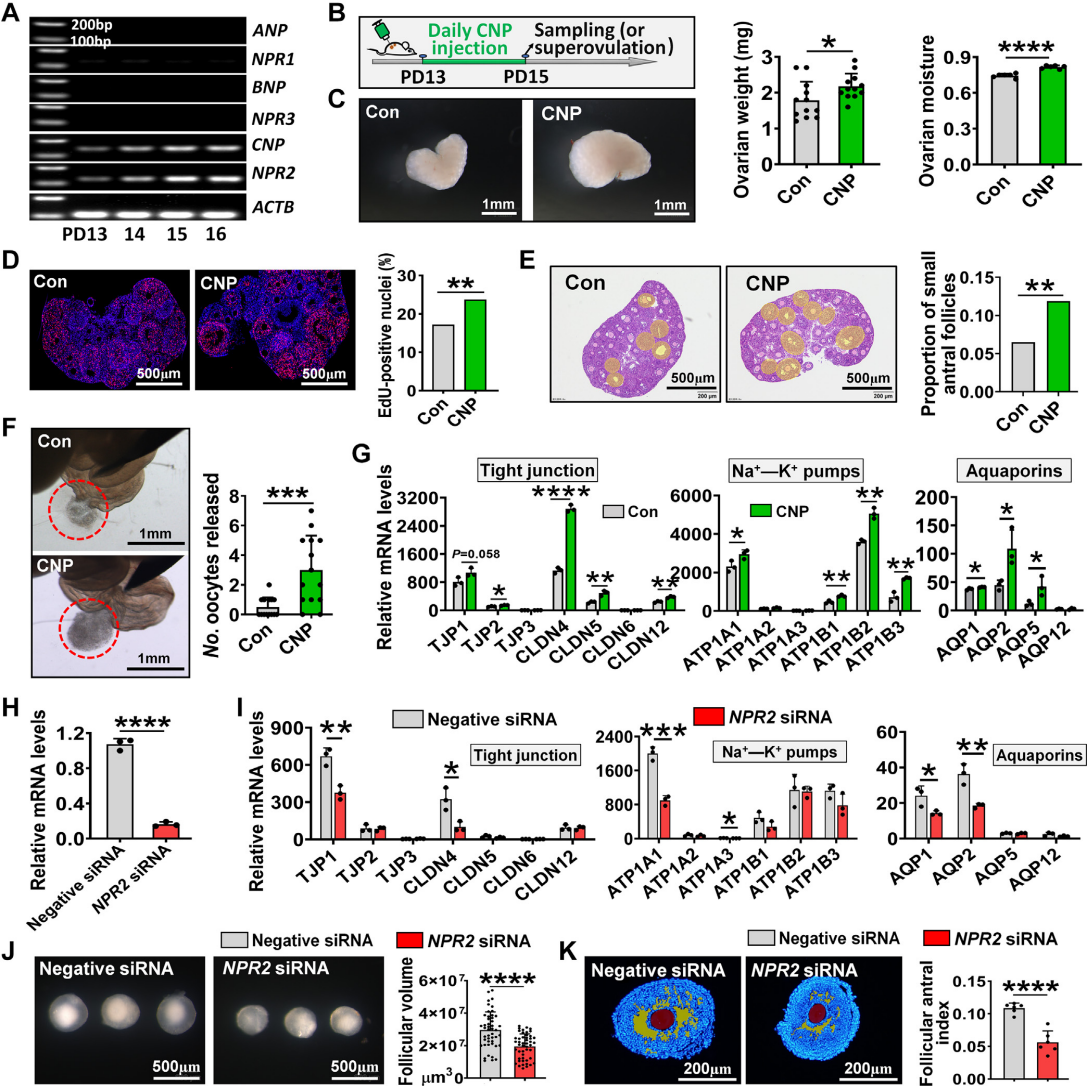
**Regulation of CNP on iFFA was related to its upregulation of tight junctions, ion pumps, and aquaporins.***A*, expression of genes in NP system during iFFA. The original bands can be viewed in Fig. S17. *B*, experimental design of CNP injection. CNP was dissolved in normal saline, and the control group was given a same dose of normal saline. *C*, effects of CNP injection on ovarian weight and moisture. *Left*, representative photographs of ovaries after CNP injection; *right*, statistics of ovarian weight (n = 12 ovaries, collected from six mice) and moisture (n = 6 biologically independent ovaries). *D*, follicular cell proliferation determined by *in vivo* EdU staining. *E*, effect of CNP injection on iFFA. *Left*, representative photographs of ovarian slices, the areas covered in *yellow* were large preantral follicles and small antral follicles; *right*, statistics of the proportion of small antral follicles, n = 6 biologically independent ovarian sections. *F*, superovulation was performed to verify the effect of CNP on iFFA. *Left*, representative photographs of oviducts, the oocytes released were surrounded by *red circles*; *right*, statistics of released oocytes, n = 14 oviducts, collected from seven mice. *G*, changes in the expression of genes encoding tight junctions, ion pumps, and aquaporins after CNP injection, n = 3 biologically independent ovaries. *H*, interference efficiency of *NPR2*, n = 3 follicular simples. *I*, changes in the expression of genes encoding tight junctions, ion pumps, and aquaporins after *NPR2* knockdown, n = 3 follicular samples. *J*, changes in follicular volume after *NPR2* knockdown, n = 47 follicles (negative siRNA) and 48 follicles (*NPR2* siRNA). *K*, changes in follicular antrum index after *NPR2* knockdown, n = 6 follicles. Statistical significance was determined using two-tailed unpaired Student’s *t* test or Chi-squared test (Fig. 4, *D* and *E*). Values are mean ± SD. ∗*p* < 0.05, ∗∗*p* < 0.01, ∗∗∗*p* < 0.001, and ∗∗∗∗*p* < 0.0001. The experiments of *C*–*F* were repeated three times, and the experiments of *G*–*K* were repeated two times; in both, similar results were obtained. CNP, C-type natriuretic peptide; iFFA, initial formation of the follicular antrum.

Based on the observation that both C-type natriuretic peptide (CNP) and its receptor *NPR2* were upregulated during iFFA, as opposed to *ANP/NPR1* and *BNP/NPR3* (Fig. 4*A*), we hypothesized that the *CNP–NPR2* pathway, which is responsible for regulating the body's water–salt balance, may be involved in iFFA. To test this speculation, mice were intraperitoneal injected with CNP daily from PD13 to 15 (Fig. 4*B*). It was observed that CNP injection significantly increased the weight and moisture content (Fig. 4*C*) of the ovaries. In line with weight alteration, EdU staining showed that the proliferation of follicular cell significantly increased after CNP injection (Fig. 4*D*). Also, the genes indicating follicular cell differentiation were upregulated after CNP injection (Fig. S8*A*). Histological analysis further showed that, compared with the control, the proportion of small antrum follicles in the CNP-treated ovaries was significantly increased (Fig. 4*E*). Consistent with histological analysis, the number of oocytes released in the CNP-treated group was significantly increased after superovulation (Fig. 4*F*). These data suggest that CNP plays a regulatory role in iFFA.

qRT–PCR analysis further confirmed that the genes encoding tight junction, ion pumps, and aquaporins were significantly upregulated in the CNP-treated ovaries compared with the control (Fig. 4*G*). Furthermore, we knocked down *NPR2* in the cultured follicles (Fig. 4*H*) and observed that the expression of genes encoding tight junction (*TJP1* and *CLDN4*), ion pumps (*ATP1A1* and *ATP1A3*), and aquaporins (*AQP1* and *AQP2*) decreased significantly after *NPR2* knockdown (Fig. 4*I*). Expectedly, the follicular volume (Fig. 4*J*), follicular antral index (Fig. 4*K*), and the expression of *CYP11A1* and *CYP19A1* (Fig. S8*B*) in cultured follicles were significantly decreased after *NPR2* knockdown compared with the control. These findings suggest that CNP signaling pathway regulates iFFA by activating tight junctions, ion pumps, and aquaporins.

### mTOR regulated iFFA by activating the CNP signaling pathway

**Figure 5:**
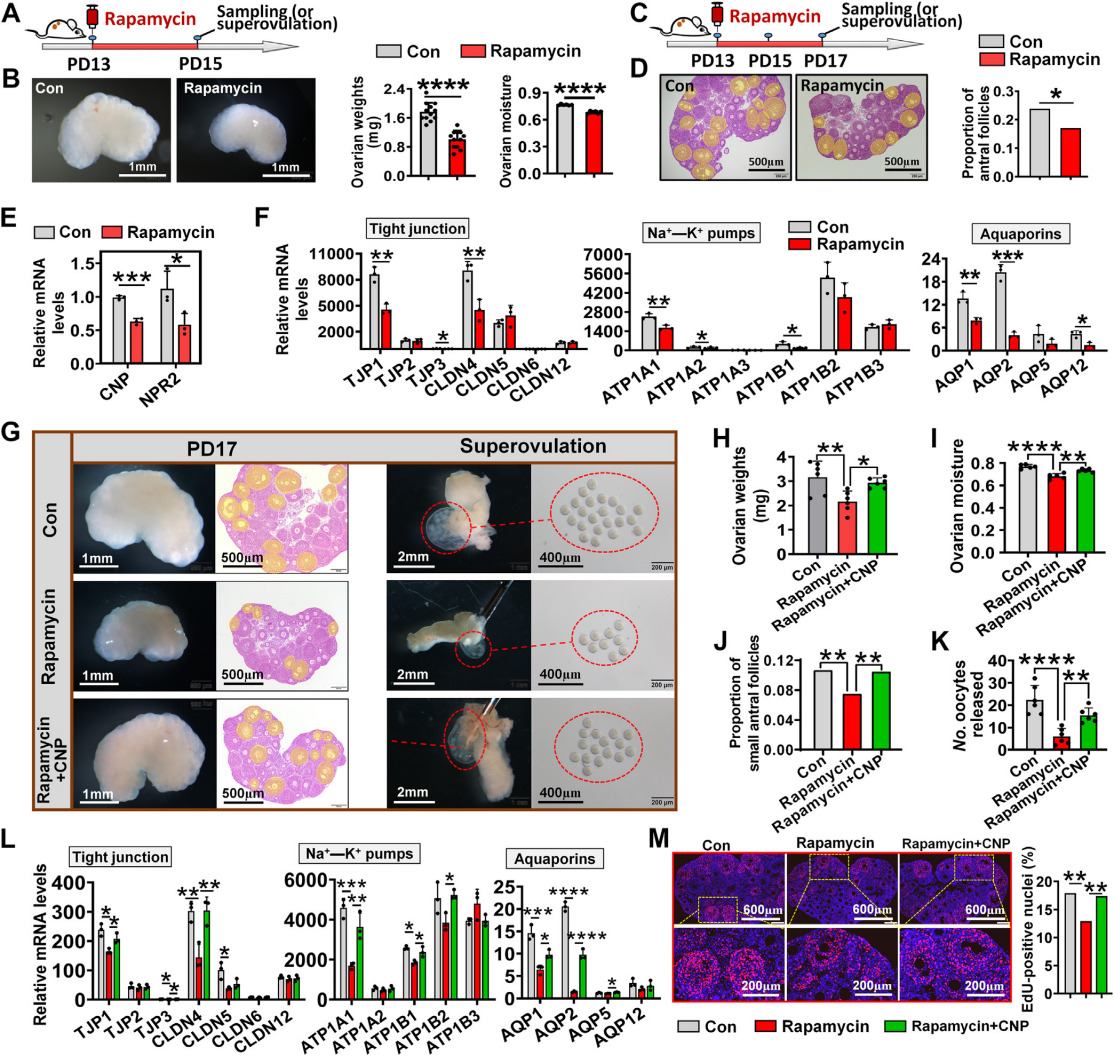
**mTOR regulated iFFA by activating the CNP signaling pathway.***A*, experimental design of *B*, *E*, and *F*; Figs. S10 and S16*B*. Rapamycin was dissolved in corn oil, and the control group received only corn oil. *B*, effect of rapamycin on ovarian weight and moisture. *Left*, representative photographs of ovaries; *right*, statistics of ovarian weight (n = 12 ovaries, collected from six mice) and moisture (n = 6 biologically independent ovaries). *C*, experimental design of Figs. 5*D* and S11. *D*, effect of rapamycin on the proportion of small antral follicles, the areas covered in *yellow* are small antral follicles, n = 6 biologically independent ovarian sections. *E*, effect of rapamycin on the expression of *CNP* and *NPR2*, n = 3 biologically independent ovaries. *F*, effect of rapamycin on the expression of genes encoding tight junctions, ion pumps, and aquaporins, n = 3 biologically independent ovaries. *G*–*K*, effects of CNP on rapamycin-induced inhibition of iFFA. In this experiment, rapamycin was given once and CNP was given daily. *G*, representative photographs of ovaries, ovarian sections, and superovulation, the areas covered in *yellow* are small antral follicles. *H*, ovarian weight, n = 6 biologically independent ovaries. *I*, ovarian moisture, n = 5 biologically independent ovaries. *J*, proportion of small antral follicles, n = 6 biologically independent ovarian sections. *K*, number of oocytes released, n = 6 biologically independent oviducts. *L*, expression of genes encoding tight junctions, ion pumps, and aquaporins, n = 3 biologically independent ovaries. *M*, follicular cell proliferation determined by *in vivo* EdU staining. Statistical significance was determined using two-tailed unpaired Student’s *t* test (Fig. 5, *B*, *E* and *F*), one-way ANOVA followed by Tukey’s post hoc test (Fig. 5, *H*–*L*), and Chi-squared test (Fig. 5, *D*, *J* and *M*). Values were mean ± SD. ∗*p* < 0.05, ∗∗*p* < 0.01, ∗∗∗*p* < 0.001, and ∗∗∗∗*p* < 0.0001. Experiments in Figure 5*M* were repeated two times, and other experiments in Figure 5 were repeated three times; similar results were obtained. CNP, C-type natriuretic peptide; iFFA, initial formation of the follicular antrum; mTOR, mammalian target of rapamycin; NPR2, natriuretic peptide receptor 2.

The mTOR signaling may play a significant role in regulating iFFA because of its activation during both iFFA and blastula cavity formation (Figs. 2*N* and 3*D*). To address this issue, rapamycin was used to inhibit mTOR. It was observed that, compared with the control, rapamycin at a dose of 5 mg/kg body weight significantly inhibited mTOR signaling at both proteic and transcriptional levels (Fig. S9). Subsequently, on PD13, mice were injected with 5 mg/kg dose of rapamycin, and their ovaries were collected on PD15 for analysis (Fig. 5*A*). The results showed that rapamycin injection significantly reduced the weight and moisture content of ovaries (Fig. 5*B*). The expression of *CYP11A1* and *CYP19A1* and the contents of estradiol and progesterone decreased significantly in the rapamycin-treated ovaries compared with the control (Fig. S10, *A* and *B*). Nevertheless, in ovaries of PD15, injection of rapamycin did not result in a significant reduction in the proportion of small antrum follicles (Fig. S10*C*) and the number of oocytes released after superovulation (Fig. S10*D*). This may be due to the fact that iFFA had just begun on PD15, and the number of small antral follicles in the ovary was not sufficient. Thus, ovaries on PD17 were collected for further analysis (Fig. 5*C*). The results showed that the small antrum follicles in rapamycin-treated group significantly also reduced (Fig. 5*D*) the oocytes after superovulation compared with the control (Fig. S11).

We speculated that the mTOR may regulate iFFA by activating the CNP signaling pathway. This hypothesis was supported by the observations of downregulation of both *CNP* and *NPR2* as well as genes encoding tight junction proteins, ion pumps, and aquaporins in rapamycin-treated group (Fig. 5, *E* and *F*). To test this speculation, CNP was injected into rapamycin-treated mice to assess whether it could rescue the inhibition of rapamycin on iFFA. The results showed that CNP injection completely reversed the effect of rapamycin on ovarian weight (Fig. 5, *G* and *H*), ovarian moisture (Fig. 5*I*), the proportion of small antral follicles (Fig. 5, *G* and *J*), and the number of oocytes released (Fig. 5, *G* and *K*). The downregulation of the genes encoding tight junction, ion pumps, and aquaporins caused by rapamycin was also reversed by CNP (Fig. 5*L*). These findings confirm that mTOR regulates iFFA by activating the CNP signaling pathway. Of note, the phosphorylation of mTOR was not affected by CNP, indicating that CNP could not impact mTOR signaling pathway through a positive feedback (Fig. S16*B*).

Rapamycin also inhibited the proliferation of follicular cells, whereas CNP restored the proliferation activity (Fig. 5*M*), suggesting that mTOR regulates follicular cell proliferation also by activating CNP signaling pathway.

### Follicle-stimulating hormone regulated iFFA by activating mTOR–CNP signaling pathway

**Figure 6:**
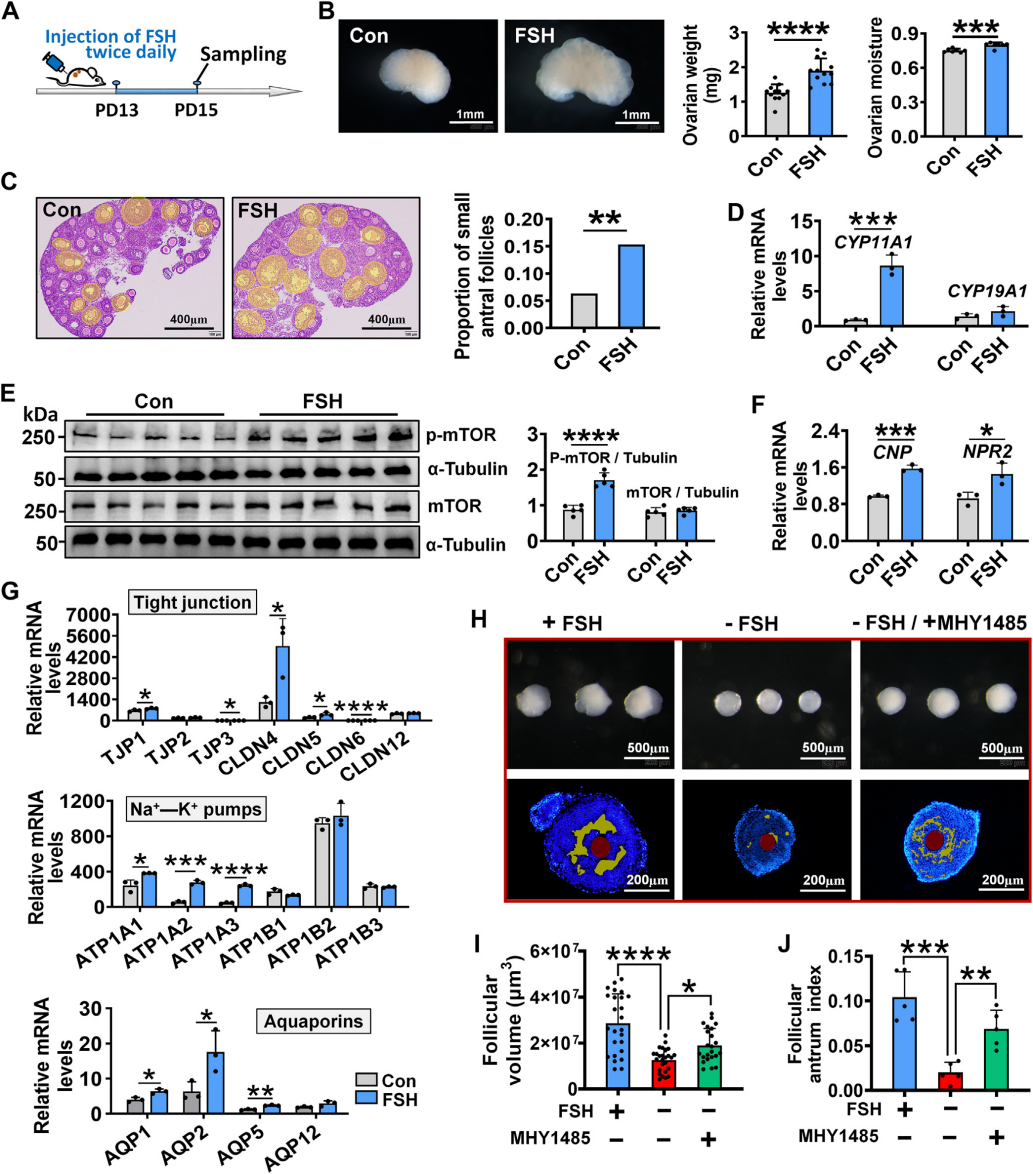
**FSH regulated iFFA by activating mTOR–CNP pathway.***A*, experimental design of FSH injection. FSH was dissolved in normal saline, and the control group was only given normal saline. *B*, effect of FSH on ovarian weight (n = 12 ovaries, collected from six mice) and moisture (n = 7 biologically independent ovaries). *C*, effect of FSH on the proportion of small antral follicles, the areas covered in *yellow* are small antral follicles. n = 5 (Con) and 6 (FSH) biologically independent sections. *D*, effect of FSH on the expression of *CYP11A1*, n = 3 biologically independent ovaries. *E*, effect of FSH on the phosphorylation of mTOR. The original blots can be viewed in Fig. S16. *F*, effect of FSH on the expression of *CNP* and *NPR2*, n = 3 biologically independent ovaries. *G*, effect of FSH on the expression of genes encoding tight junctions, ion pumps, and aquaporins, n = 3 biologically independent ovaries. *H*–*J*, MHY1485 (MCE) rescued iFFA blockage caused by FSH absence. *H*, representative photographs of follicles and follicular slices. MHY1485 was dissolved in DMSO, culture medium containing 1% DMSO was used as the control. *I*, follicular volume, n = 25 follicles (FSH^+^ MHY1485^−^), 26 follicles (FSH^−^ MHY1485^−^), and 23 follicles (FSH^−^ MHY1485^+^). *J*, follicular antrum index, n = 5 follicles. Statistical significance was determined using two-tailed unpaired Student’s *t* test (Fig. 6, *B*, *D*, *E*, *F* and *G*), one-way ANOVA followed by Tukey’s post hoc test (Fig. 6, *I* and *J*), and Chi-squared test (Fig. 6*C*). Values are mean ± SD. ∗*p* < 0.05, ∗∗*p* < 0.01, ∗∗∗*p* < 0.001, and ∗∗∗∗*p* < 0.0001. *B*–*G*, the experiments were repeated three times, and the experiments in *H*–*J* were repeated two times; in both, similar results were obtained. CNP, C-type natriuretic peptide; DMSO, dimethyl sulfoxide; FSH, follicle-stimulating hormone; iFFA, initial formation of the follicular antrum; mTOR, mammalian target of rapamycin; *NPR2*, natriuretic peptide receptor 2.

We continued to investigate the signaling controlling the intraovarian mTOR–CNP signaling pathway. Based on both RNA-Seq and qRT–PCR data, which indicated an upregulation of the *FSHR* during iFFA (Fig. S12, *A* and *B*), we hypothesized that the extraovarian hormone FSH activates mTOR–CNP. This hypothesis is also supported by a previous report that *Fsh-β* deletion caused folliculogenesis to be blocked in the preantral stage (45). To test this hypothesis, mice were injected with FSH twice daily from PD13 to 15 (Fig. 6*A*). Results showed that the injection of FSH significantly increased the weight and moisture content of ovaries (Fig. 6*B*). Histological analysis also revealed an increase in the proportion of small antrum follicles in FSH-treated ovaries compared with the control (Fig. 6*C*). Moreover, the expression of *CYP11A1* (Fig. 6*D*) and genes regulating proliferation (Fig. S13) significantly increased following FSH injection (Fig. S13). Furthermore, the increased phosphorylation of mTOR (Fig. 6*E*), upregulation of genes in CNP system (*CNP* and *NPR2*) (Fig. 6*F*), tight junction (*TJP1*, *TJP3*, *CLDN4*, *CLDN5*, and *CLDN6*), ion pumps (*ATP1A1*, *ATP1A2*, and *ATP1A3*), and aquaporins (*AQP1*, *AQP2*, and *AQP5*) (Fig. 6*G*) were observed with FSH injection. Finally, we tested whether MHY1485, an mTOR activator, could rescue the iFFA blockage caused by FSH absence. Specifically, preantral follicles were isolated and cultured, and iFFA was assessed at 120 h of culture. In comparison to the control group, the absence of FSH resulted in a decrease in follicular volume and follicular antrum index, whereas supplementation with 5 μM MHY1485 restored these traits (Fig. 6, *H*–*J*).

These results suggest that extraovarian FSH regulates iFFA by activating the intraovarian mTOR–CNP signaling pathway. Notably, we observed that rapamycin injection did not affect the expression of *FSHR* in the ovary (Fig. S12*C*). This may indicate that mTOR–CNP is unable to enhance FSH signaling through positive feedback.

### Short-term MHY1485 treatment promoted iFFA and oocyte yield *in vitro*

**Figure 7:**
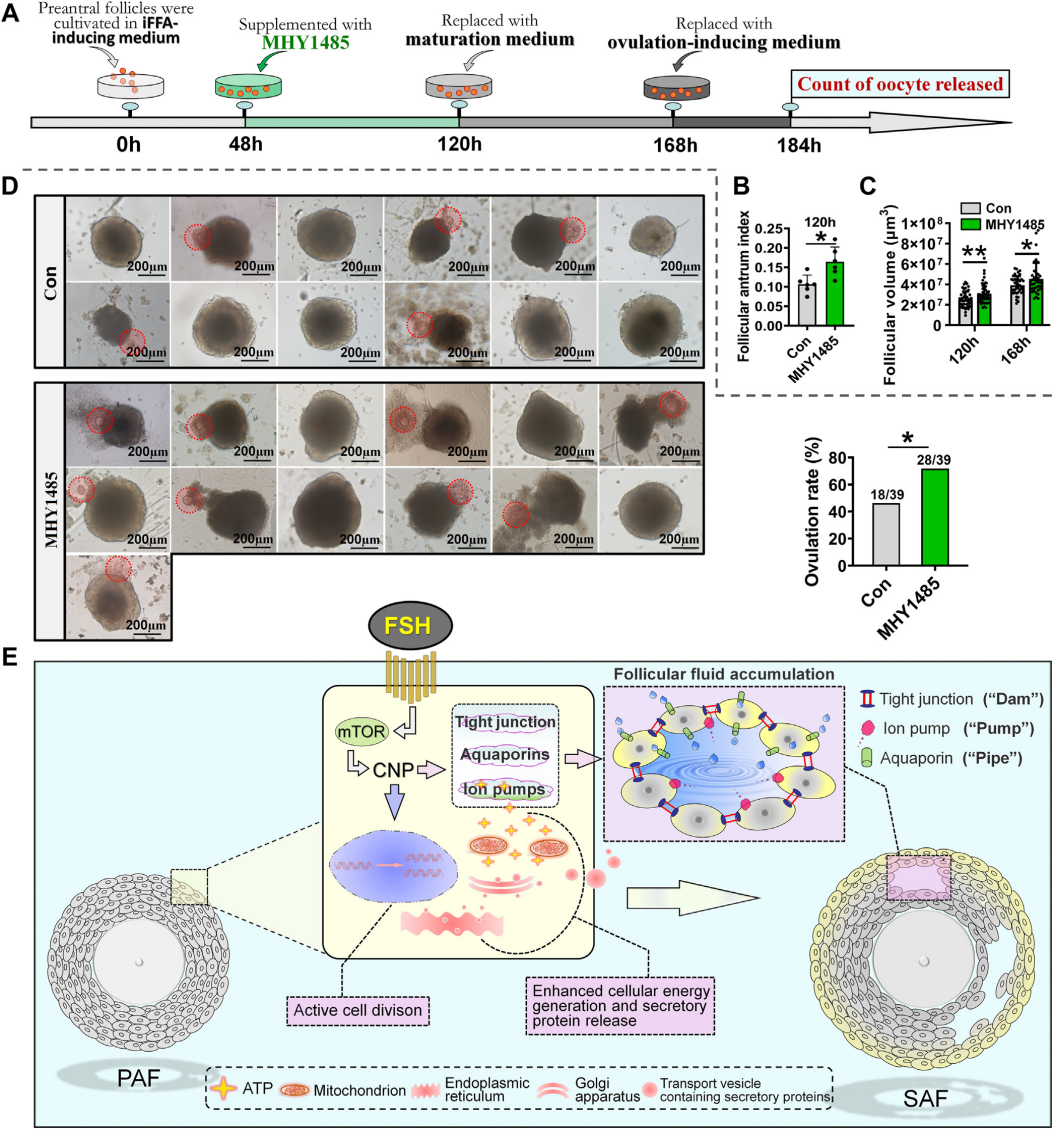
**Short-term MHY1485 treatment promoted iFFA and oocyte yield *in vitro*.***A*, experimental protocol for follicle culture with MHY1485. MHY1485 was only added into iFFA-inducing medium but not into maturation and ovulation-inducing medium. *B*, effect of MHY1485 on follicular antrum index, n = 6 follicles. *C*, effect of MHY1485 on follicular volume, n = 44 (120 h/Con), 40 (120 h/MHY1485), and 39 (168 h) follicles. *D*, effect of MHY1485 on ovulation rate. *Left*, representative photographs of follicles after ovulation induction; *right*, statistics of ovulation rate, n = 39 follicles. *E*, a diagram summarizing the regulatory mechanism of iFFA. FSH enhances the mTOR–CNP signaling pathway in follicular cells. This pathway stimulates cell proliferation, leading the follicle's growth to the appropriate size for iFFA to occur. Simultaneously, it triggers the “Dam–Pump–Pipe” mechanism, promoting fluid flow into the follicle, thus resulting in the formation of small antra within the follicle. In addition, cellular energy generation is enhanced during iFFA, which supports the efficient operation of the pumps. Moreover, the increase in the synthesis of large macromolecule hydrophilic secretory proteins may also contribute to the elevation of the osmotic pressure gradient, aiding to the pumps. Statistical significance was determined using two-tailed unpaired Student’s *t* test (Fig. 7, *B* and *C*) and Chi-squared test (Fig. 7*D*). Values are mean ± SD. ∗*p* < 0.05 and ∗∗*p* < 0.01. The experiment was repeated three times independently, and similar results were obtained. CNP, C-type natriuretic peptide; FSH, follicle-stimulating hormone; iFFA, initial formation of the follicular antrum; mTOR, mammalian target of rapamycin; PAF, preantral follicle; SAF, small antral follicle.

The technology of follicular activation and culture *in vitro* has been developed, which has successfully delivered healthy babies to patients with premature ovarian insufficiency (46, 47). Figure 5 illustrated that mTOR regulates iFFA by activating CNP*–*NPR2 pathway. Therefore, we wondered whether MHY1485, an mTOR activator, could promote iFFA and then increased oocyte yield in follicular culture technology. To address this, preantral follicles were isolated from the ovaries for culture, and MHY1485 was added to the iFFA-inducing medium from 48 h to 120 h (Fig. 7*A*). After 120 h of culture, follicular antrum index and follicular volume were measured. The results showed that MHY1485 treatment significantly increased the follicular antrum index (Fig. 7*B*) and the follicular volume (Fig. 7*C*). The ovulation rate in MHY1485 group was also significantly higher than that in the control group (Fig. 7*D*). These results indicate that the mTOR activator could be used as an enhancer to increase the oocyte yield in *in vitro* follicular culture, which is of clinical significance.

## Discussion

As mentioned previously that the studies on iFFA are lagged behind other aspects of folliculogenesis, and this negatively impact our understanding on animals including human reproductive physiology. In this study, we have attempted to elucidate the potential mechanism underlying iFFA by use of different molecular technologies. We found that iFFA shares a regulatory mechanism with blastula cavity formation and is characterized by enhanced fluid absorption, energy consumption, secretion, and proliferation. Most importantly, we proposed a “Dam–Pump–Pipe” model to explain the mechanism of follicular fluid formation during iFFA and revealed that the FSH–mTOR–CNP signaling axis plays a vital role in triggering “Dam–Pump–Pipe.”

According to the “Dam–Pump–Pipe” model (Fig. 7*E*), tight junction forms a sealed compartment within the follicle, acting as a “Dam” to maintain the osmotic pressure gradient inside the follicle. Ion pumps, especially the Na^+^–K^+^ pumps, are responsible for generating the pressure gradient and function as the “kinetic pump” to propel follicular fluid. Aquaporins facilitate fluid transport across the follicular cells and act as the “pipe” that directs the fluid flow into the follicular antrum. The “Dam–Pump–Pipe” model not only offers a reasonable theoretical explanation for iFFA but also helps in further understanding of follicular antrum formation from different perspectives. For instance, the perspective that there were no tight junctions among follicular cells (18, 25) might be incorrect. The “Dam–Pump–Pipe” model suggests that tight junctions form within the follicle, at least during the iFFA phase. A knockdown of the tight junction's core structural gene inhibits iFFA. The existence of tight junctions within the follicle might also evidence the presence of a blood–testis barrier–like structure that protects oocyte within follicle. This is because blood–testis barrier, consisting of the tight junctions, is a physical barrier that protects sperm in the seminal tubules (48). The traditional view that hydrophilic macromolecules solely create the osmotic pressure gradient driving follicular fluid absorption (18, 25) may also need to update since the “Dam–Pump–Pipe” model proposes that ions, particularly Na^+^, may be the primary agents in generating the osmotic pressure. This is supported by the fact that knocking down *ATP1A1*, which encodes a Na^+^–K^+^ ATPase pump, reduces the size of the follicular antrum by half (Fig. 3*K*). Therefore, the increased energy expenditure during iFFA (Fig. 2, *H*–*N*) may be due to the necessity of facilitating the efficient operation of ion pumps. However, it remains unclear to what extent ion pumps and hydrophilic macromolecules contribute to the osmotic pressure gradient in the follicle, respectively.

FSH–mTOR–CNP is a key signaling pathway uncovered to trigger “Dam–Pump–Pipe” mechanism, resulting in the onset of iFFA. The discovery of this pathway suggests that the responsiveness of follicular cells to FSH is a prerequisite for iFFA. Therefore, although it is still unclear what the starting signals for iFFA is, it is definitely the signal that upregulates *FSHR*. We speculate that FSH–mTOR–CNP is a unidirectional pathway. This is supported by the fact that FSH increased mTOR phosphorylation, whereas inhibiting mTOR phosphorylation had no effect on *FSHR* abundance (Figs. 6*E* and S12*C*). Furthermore, mTOR activated CNP pathway, but administering CNP did not result in increased mTOR phosphorylation (Figs. 5*E* and S16*B*).

The discovery of FSH–mTOR–CNP signaling pathway has practical applications. First, it could improve the follicle culture outcomes by activating mTOR as we did in this study (Fig. 7). Second, it provides a theoretical foundation for improving superovulation protocols—a core technology for test-tube animal production and human-assisted reproduction. The number of oocytes obtained through superovulation is directly proportional to the number of small antral follicles in the ovary. This is because only antral follicles respond to gonadotropins for mature and ovulation (43, 49). Thus, before superovulation, augmenting the FSH–mTOR–CNP pathway with some agents can be an effective strategy to increase the yield of oocytes. Notably, the mTOR–CNP may not be the only signaling pathway for iFFA since the enhanced energy production during iFFA was not impaired by mTOR inhibition (Fig. S14). Other unknown signaling pathways may also be responsible for enhancing energy metabolism during iFFA.

Our study also provides new insights into the function of CNP in early folliculogenesis. Over the past decade, a couple of studies have reported the potential effects of CNP on the growth of preantral follicle and the number of small antral follicles (50, 51). However, no in-depth exploration has been conducted regarding the mechanism. We confirmed that CNP has a strong promotion effect on follicular cell proliferation, which is most likely the cause for its ability to promote preantral follicle growth. Furthermore, our findings highlight that CNP can induce iFFA by upregulating the expression of genes encoding tight junction, ion pumps, and aquaporins. This effect could still be observed in situations where proliferation was blocked completely by urolithin A (MCE) (Fig. S15). Based on these observations, we could propose a reasonable conjecture on the role of CNP in early folliculogenesis, that is, CNP promotes cell proliferation for follicle's growth to the appropriate size ready for iFFA, and then triggers the “Dam–Pump–Pipe” mechanism, resulting in the occurrence of iFFA.

Notably, for the roles of CNP and *NPR2* in folliculogenesis, some conflicting findings have been reported. For example, Sato *et al.* (50) and Xi *et al.* (51) reported the promoting effect of CNP on antral follicle formation. This was supported by the observations of Tamura *et al.* (52), in which the absence of antral follicles in the ovary was caused by *NPR2* knockout. Conversely, Geister *et al.* (53), Kiyosu *et al.* (54), and Zhang *et al.* (55) observed normal folliculogenesis in ovaries with spontaneous mutations of *NPR2*. Our results showed that *NPR2* knockdown hinders follicular antrum formation. To address this obvious conflict results, we gathered information on existing *NPR2* mutant strains from MGI database. After comparing the phenotypes of these strains, we observed significant differences in their reproductive performance. For instance, in the knockout mutant strain of *NPR2*^tm1Gar^, with a genetic background of 129S6/SvEvTac ∗ C57BL/6, females showed infertility because of the absence of oestrous cycles, whereas males can be fertile if they survive (52). On the other hand, in the spontaneous mutant strains, such as the type of *NPR2*^pwe^ with a genetic background of C3H/HeJ ∗ C57BL/6 ∗ NAW/WI, females displayed normal estrous cycles but were infertile because of their oocytes undergoing abnormal meiosis, whereas males were subfertile (53). Conversely, in the *NPR2*^cn-3J^ type with a background of MRL/MpJ-Npr2^cn-3J^/GrsrJ, females experience subfertile, whereas males are unable to breed. As for the *NPR2*^cn^ type with a background of AKR/J, both male and female homozygotes are subfertile (56). Therefore, it is our speculation that discrepancies in mutation strains and genetic backgrounds of the experimental mice may have caused the inconsistency mentioned previously.

To summarize, this study proposed a “Dam–Pump–Pipe” model to shed light on the mechanism by which follicular fluid enters the antra during iFFA (Fig. 7*E*) and revealed that the FSH–mTOR–CNP signaling axis regulates iFFA by triggering “Dam–Pump–Pipe.” These findings represent an advancement in the research of follicular antrum formation and contribute to a deeper understanding of folliculogenesis.

### Limitations of this study

(1) Although this study has accomplished efficient knockdown of *CLDN4*, *ATP1A1*, *AQP2*, and *NPR2* in the cultured follicles, conditional knockouts were not conducted, thereby limiting the assessment of the effect of these gene deletions on the phenotype of the individuals. (2) Numerous isoforms of ion pumps and aquaporins are expressed in follicles, whereas only highly expressed *ATP1A1* and *AQP2* were knocked down in this study, which means that the ion pumps and water channels were not entirely disrupted. (3) Because of the challenging nature of isolating follicles during the transition from preantrum to antrum, whole ovaries within the time frame of iFFA were used for *in vivo* experiments, which may have introduced interference from other ovarian constituents. To boost the dependability of this study, the experiments were independently repeated three times, and *in vitro* trials were conducted to corroborate *in vivo* findings.

## Experimental procedures

### Animals

Kunming mice were reared in a specific pathogen–free laboratory animal house and kept at a constant temperature of 22 ± 2 °C, with 12 h light–dark cycles (light service from 7:00 to 19:00) and allowed access to food and water *ad libitum*. All experiments and animal handling were conducted in accordance with the guidelines of animal experimental institutions after obtaining the prior approval from the Institutional Animal Ethics Committee of Huazhong Agricultural University (HZAUMO-2020-0082).

### RNA-Seq and bioinformatics analysis

To determine the dynamic changes of transcriptome during iFFA, the ovaries were collected on PD14 to 16 and lysed using TRIzol reagent (Invitrogen) for RNA extraction. RNA-Seq and data annotation were completed by NOVOGENE. Briefly, a total amount of 1 μg RNA per sample was used as input material for the RNA sample preparations. Sequencing libraries were generated using NEBNext Ultra RNA Library Prep Kit for Illumina (NEB) following the manufacturer’s recommendations. After quality inspection, the library preparations were sequenced on an Illumina Hiseq platform (NEB). Gene expression was normalized as the fragments per kilobase of exon per million fragments mapped (FPKMs), and the genes with an FPKM >1 were defined as expressed genes. The differential expression between time points was assessed by principal component analysis (NEB). Furthermore, the average FPKM value of genes at each time point was imported into STEM software (School of Computer Science, Carnegie Mellon University) to generate gene expression clusters. Genes in upregulated expression cluster were then imported into DIVAD database for GO analysis (https://david.ncifcrf.gov/tools.jsp). The upregulated secretory protein–encoding genes were derived from the intersection of human secretory protein–encoding genes, homologous genes between human and mice, and the upregulated gene expression cluster. Human and mouse homologous genes were downloaded from the Vertebrate Homology Database (http://www.informatics.jax.org/homology.shtml). The upregulated secretory protein–encoding genes were then imported into KOBAS database for Kyoto Encyclopedia of Genes and Genomes analysis (http://kobas.cbi.pku.edu.cn/home.d).

Embryo transcriptome data were obtained from Gene Expression Omnibus database (accession number: GSE128691). Only genes with FPKM >1 were defined as expression genes. Next, the genes upregulated during blastocele formation were generated by STEM and intersected with the upregulated genes during iFFA. Finally, genes in the intersection were subjected to GO analysis to identify the common regulatory factors between iFFA and blastocoel formation.

### Intraperitoneal injection

Mice were given drugs by intraperitoneal injection, and the number of injections was determined by drug stability. Briefly, CNP (Sigma) and FSH (Ningbo Secondary Hormone Co, Ltd) were diluted into working fluid with sterile normal saline. Rapamycin (MCE) was diluted into working fluid with corn oil (MCE) prior to injection. To determine the effect of CNP on iFFA, mice were injected with CNP at a dose of 100 μg/kg (body weight) daily from PD13 to PD15. To determine the effect of FSH on iFFA, mice were injected with FSH (2 IU/mouse) at 12 h intervals from PD13 to PD15. To determine the effect of rapamycin on iFFA, mice were injected with rapamycin (2 mg or 5 mg/kg) once on PD13. To assess whether CNP could rescue the inhibition of rapamycin on iFFA, mice were injected with rapamycin (5 mg/kg) once at PD13, whereas CNP injection (100 μg/kg) was given daily from PD13 to PD15.

### Superovulation

Mice on PD15 or 17 were injected with 5 IU pregnant mare serum gonadotropin (Ningbo Sansheng Biological Technology) to stimulate follicle growth to the preovulatory stage. About 48 h after pregnant mare serum gonadotropin injection, human chorionic gonadotropin (Ningbo Sansheng Biological Technology) at a dose of 5 IU was injected to trigger ovulation. After an additional 14 h, the oocytes were harvested from oviducts and counted.

### Follicle culture

Follicles were placed in 96-well plates (BKMAM) covering with mineral oil (Sigma) and cultured in an incubator (5% CO_2_, 37 °C, and saturated humidity). Briefly, small preantral follicles with diameters ranging from 100 to 120 μm were isolated from ovaries using 33-gauge microneedles (KONSFI and cultured in iFFA-inducing medium. The iFFA-inducing medium used alpha minimum essential medium (ɑ-MEM) (Gibco) as the basic solution and supplemented with 1% ITS (Sigma), 5% fetal bovine serum (FBS) (ExCell Bio), 10 mIU/ml FSH (GLPBIO), 10 mIU/ml luteinizing hormone (Sigma), 100 U/ml penicillin–streptomycin (Solarbio). After 120 h of culture, the follicles were transferred into maturation medium. The maturation medium used ɑ-MEM as the basic solution and supplemented with 1% ITS, 5% FBS, 100 mIU/ml FSH, 10 mIU/ml luteinizing hormone, 100 U/ml penicillin–streptomycin, and 100 pg/ml androstenedione (Sigma). Two days later, the mature follicles were transferred into ovulation-inducing medium to trigger ovulation. The ovulation-inducing medium also used ɑ-MEM as the basic solution and supplemented with 1% ITS, 5% FBS, 100 mIU/ml FSH, 10 IU/ml human chorionic gonadotropin, 10 ng/ml epidermal growth factor (Sigma), and 100 U/ml penicillin–streptomycin.

To completely block follicular cell proliferation, follicles with diameters ranging from 170 to 200 μm were randomly placed in iFFA-inducing medium either containing 15 μM urolithin A or lacking it. To evaluate whether CNP can induce iFFA in the presence of proliferation inhibition, a dose of 200 ng/ml CNP was added to the medium 12 h after urolithin A was added. The duration of the proliferation inhibition experiment was 36 h, and the iFFA-inducing medium used did not contain FSH.

### RNA interference

Lentivirus-mediated RNA interference was used to inhibit the expression of target genes in follicles. Briefly, PLKO.1-EGFP-PURO plasmid (GeneCreate) was used to construct interference vectors, siRNAs targeting *CLDN4*, *ATP1A1*, *AQP2*, and *NPR2* were synthesized by Genepharma (targeted sequence-*CLDN4*: 5′-gcgacttctacaaccctatgg-3′; targeted sequence-*ATP1A1*: 5′-ggatgagctcaagaaggaagt-3′; targeted sequence-*AQP2*: 5′-ggttccctcctctacaactac-3′; and targeted sequence-*NPR2*: 5′-gcagctaagaatgagcattat-3′. The scrambled shRNA was used as negative siRNA control. pMD2.G and pSPAX were purchased from GeneCreate. Lentiviruses were produced in 293 T cells (ATCC) by cotransfecting 4.8 μg interference vector, 2.4 μg pMD2.G, and 4.8 μg pSPAX2. About 48 h later, the viral supernatants were harvested, centrifuged, and filtered with 0.45 μm polyvinylidene fluoride membranes (Sigma). Next, the follicles with diameters ranging from 100 to 120 μm were placed in iFFA-inducing medium and transfected with the prepared viral supernatants (titer: 1.25 × 10^7^ viral particles/ml) using Polybrene (Sigma). The transfection lasted for 48 h. After transfection, the follicles were cultured in fresh iFFA-inducing medium for 72 h and then collected for determination of interference efficiency, follicular volume, and follicular antrum index. To inhibit the expression of target gene in the ovary, mice of PD13 were anesthetized, and the ovaries were exposed *via* a dorsal incision. Finally, the lentiviral particles (10 μl, titer: 1.25 × 10^9^ viral particles/ml) were injected beneath the ovarian bursa.

### H&E staining

Ovaries were collected according to experimental requirements and then fixed in 4% paraformaldehyde (Servicebio). After paraffin embedding, the ovaries were sectioned into slices (5 μm thick) along the longitudinal axis. After deparaffinization and rehydration, the slices were stained with eosin and hematoxylin (Servicebio) for 5 min each. Thereafter, the dyed slices were dehydrated with gradient alcohol, transparented with xylene, and sealed with mounting medium. Photographs were acquired from a microscope (Olympus). Finally, the follicular area was measured using ImageJ software (National Institutes of Health), the numbers of growing follicles, and small antral follicles were counted to calculate the proportion of small antral follicles.

### EdU staining

EdU assay kit was purchased from RiboBio. The dose of EdU used for intraperitoneal injection in this study was 5 mg/kg. To determine the changes of follicular cell proliferation during iFFA, mice were injected with EdU on PD14, 15, and 16. To determine the effect of CNP on follicular cell proliferation, mice were injected with EdU at PD15. Ovaries were collected 24 h after EdU injection and made into paraffin slices (5 μm thick). Finally, the slices were incubated with EdU dye reaction solution for 30 min, and the nuclei were stained with 4′,6-diamidino-2-phenylindole (Servicebio) for 10 min. Photographs were taken and analyzed using a fluorescence microscope (Olympus). EdU-positive nuclei emitted red fluorescence, and the proliferation of follicular cells was measured by counting the proportion of red fluorescence nuclei.

### Immunofluorescence

Immunofluorescence was used to detect the formation of tight junction in follicles. Briefly, ovaries on PD15 were collected and sectioned (5 μm thick). After deparaffinized and rehydrated, the slices were incubated with antigen retrieval buffer (Solarbio) at 98 °C and then blocked with 3% bovine serum albumin (Servicebio) for 30 min. Next, the sections were incubated with the primary antibody ZO-1 (1:200 dilution; Bioss) for 16 h at 4 °C. After washing, the slices were incubated with FITC-conjugated secondary antibody (1:200 dilution; ABclonal) for 60 min at room temperature. Finally, the slices were stained with 4′,6-diamidino-2-phenylindole (Biosharp) and sealed in the antifade fluorescence mounting medium (Servicebio). Photographs were taken and analyzed using a laser scanning confocal microscope (Carl Zeiss AG).

### Frozen section

To determine follicular antral index, the cultured follicles were taken out for frozen section. Briefly, the follicles were transferred to an optimum cutting temperature compound (Sakura)–covered embedding box for embedding. The embedded follicles were then plunged into liquid nitrogen for quick freezing and taken out after 1 min. Thereafter, the frozen follicles were sectioned (6 μm thick) using a frozen microtome (Leica) and stained with 4′,6-diamidino-2-phenylindole. Photographs were taken and analyzed using a fluorescence microscope (Olympus). The follicular area and follicular antrum area were measured using ImageJ software to calculate follicular antrum index (follicular antrum index = follicular antrum area/follicular area).

### PCR analysis

Total RNA was extracted using the Trizol reagent. Reverse transcription was performed using the PrimeScript RT reagent kit (Takara). The real-time qPCR was performed using a CFX384 Real-Time PCR System (Bio-Rad). The reaction system contained 5 μl SYBR Green (Biosharp), 2 μl template complementary DNA, forward and reverse primers (250 nM for each), and ddH_2_O was added up to a total volume of 10 μl. The reaction procedure was as follows: predegeneration 95 °C for 10 min; 35 cycles of denaturation at 95 °C for 10 s, and annealing/extension at 60 °C for 30 s; 95 °C for 10 s; and melting curve from 60 °C to 95 °C, increasing in an increment of 0.5 °C every 5 s. Normalization was performed using the housekeeping gene *ACTB*. Relative RNA quantification was performed *via* the comparative 2^−△△Ct^ method. RT–PCR was run using a PCR Instrument (Scilogex). The reaction system contained 10 μl 2× Taq Plus PCR MasterMix (Tiangen Biotech), 4 μl template complementary DNA, forward and reverse primers (250 nM for each), and ddH_2_O was added up to a total volume of 20 μl. The reaction procedure was as follows: predenaturation at 95 °C for 10 min; 35 cycles of denaturation at 95 °C for 30 s, annealing at 60 °C for 30 s, extension at 72 °C for 30 s; and a final elongation step at 72 °C for 5 min. The amount of PCR products was determined by agarose gel electrophoresis (120 V, 100 mA, 60 min). The primers are listed in Table 1.

**Table 1:** The primers used for qPCR and RT–PCR

Gene	Primer sequence (5′-3′)	Product size (bp)
*FSHR*	Forward: TGCTCTAACAGGGTCTTCCTC	84
	Reverse: TCTCAGTTCAATGGCGTTCCG	
*CYP11A1*	Forward: AGGTCCTTCAATGAGATCCCTT	137
	Reverse: TCCCTGTAAATGGGGCCATAC	
*CYP19A1*	Forward: AACCCCATGCAGTATAATGTCAC	132
	Reverse: AGGACCTGGTATTGAAGACGAG	
*AQP1*	Forward: AGGCTTCAATTACCCACTGGA	124
	Reverse: GTGAGCACCGCTGATGTGA	
*AQP2*	Forward: ATGTGGGAACTCCGGTCCATA	137
	Reverse: ACGGCAATCTGGAGCACAG	
*AQP5*	Forward: AGAAGGAGGTGTGTTCAGTTGC	220
	Reverse: GCCAGAGTAATGGCCGGAT	
*AQP12*	Forward: CAGCTCAACCCTGCGTACATC	154
	Reverse: TGTGTAGGCCATGAGAGTGAC	
*ATP1A1*	Forward: GGGGTTGGACGAGACAAGTAT	173
	Reverse: CGGCTCAAATCTGTTCCGTAT	
*ATP1A2*	Forward: CCACCACTGCGGAAAATGG	143
	Reverse: GCCCTTAGACAGATCCACTTGG	
*ATP1A3*	Forward: TCTCAGATGTGTCCGTTCTTCT	65
	Reverse: TGGAAAGAGAGTGAAAGGCAAG	
*ATP1B1*	Forward: GCTGCTAACCATCAGTGAACT	115
	Reverse: GGGGTCATTAGGACGGAAGGA	
*ATP1B2*	Forward: GGCAGGTGGTTGAGGAGTG	181
	Reverse: GGGGTATGGTCAGAGACGGT	
*ATP1B3*	Forward: CCGAGCAGCGGAGAGTTTC	139
	Reverse: CTTCATCATTCAGAGTCTGGAGC	
*ATP4A*	Forward: GATGGAGATTAACGACCACCAG	169
	Reverse: ACGGGCAAACTTCACATACTC	
*ATP4B*	Forward: CAGGAGAAGAAGTCATGCAGC	129
	Reverse: GAAACCTGCGTAGTACAGGCT	
*TJP1*	Forward: GCCGCTAAGAGCACAGCAA	134
	Reverse: TCCCCACTCTGAAAATGAGGA	
*TJP2*	Forward: ATGGGAGCAGTACACCGTGA	176
	Reverse: TGACCACCCTGTCATTTTCTTG	
*TJP3*	Forward: TCGGCATAGCTGTCTCTGGA	194
	Reverse: GTTGGCTGTTTTGGTGCAGG	
*CLDN4*	Forward: GTCCTGGGAATCTCCTTGGC	112
	Reverse: TCTGTGCCGTGACGATGTTG	
*CLDN5*	Forward: GCAAGGTGTATGAATCTGTGCT	109
	Reverse: GTCAAGGTAACAAAGAGTGCCA	
*CLDN6*	Forward: ATGGCCTCTACTGGTCTGCAA	215
	Reverse: GCCAACAGTGAGTCATACACCTT	
*CLDN12*	Forward: TGTCCTTCCTGTGTGGTATTGC	117
	Reverse: AAATCGTCAGGTTCTTCTCGTTT	
*OCLN*	Forward: TTGAAAGTCCACCTCCTTACAGA	129
	Reverse: CCGGATAAAAAGAGTACGCTGG	
*ANP*	Forward: GCTTCCAGGCCATATTGGAG	126
	Reverse: GGGGGCATGACCTCATCTT	
*BNP*	Forward: GAGGTCACTCCTATCCTCTGG	100
	Reverse: GCCATTTCCTCCGACTTTTCTC	
*CNP*	Forward: GGTCTGGGATGTTAGTGCAGCTA	139
	Reverse: TAAAAGCCACATTGCGTTGGA	
*NPR1*	Forward: ATCCCAGACCGCTCATGGA	120
	Reverse: TCGACGAACTCCTGGTGATTTA	
*NPR2*	Forward: TGCTGCCAGAACACAACCTG	122
	Reverse: TTCGGAGCTGACAAACCGC	
*NPR3*	Forward: GTCTACAGCGACGACAAACTC	125
	Reverse: AGGTCCAAGTCTTTGGTCTCG	
*SLC2A1*	Forward: CAGTTCGGCTATAACACTGGTG	156
	Reverse: GCCCCCGACAGAGAAGATG	
*SLC2A8*	Forward: CCCTTCGTGACTGGCTTTG	138
	Reverse: TGGGTAGGCGATTTCCGAGAT	
*SLC2A10*	Forward: GCCTGACCTTCGGATATGAGC	165
	Reverse: TGCCATAGCAGTCAATGAGGA	
*mTOR*	Forward: CAGTTCGCCAGTGGACTGAAG	130
	Reverse: GCTGGTCATAGAAGCGAGTAGAC	
*MLST8*	Forward: CCAATAACCCCAACCCCAT	107
	Reverse: GCAGTCCTCGCCACCTGTA	
*RAPTOR*	Forward: GTGGAAAACAGGAGCCCCC	103
	Reverse: CCAAAAACCGTCCCAGCAA	
*PRAS40*	Forward: CTGCTCCTAGTCCACCACCT	123
	Reverse: AGAGACCTCCATTATCGCTACC	
*DEPTOR*	Forward: ATAGACGGCACCATCTCAAAAC	128
	Reverse: GTCGGCTAATTTCTGCATGAGT	
*ACTB*	Forward: CCAGCCTTCCTTCTTGGGTAT	182
	Reverse: AGGTCTTTACGGATGTCAACG	
*CYCLIN B*	Forward: GCTGGTCGGTGTAACGGC	195
	Reverse: GATGCTCTACGGAGGAAGTGC	
*CYCLIN D1*	Forward: GCTGGTAGTATGAGGTGCTTGG	86
	Reverse: CTTTGCAGGACAGATCCCG	
*P21*	Forward: TTCCGCACAGGAGCAAAG	130
	Reverse: ACGAAGTCAAAGTTCCACCG	
*P27*	Forward: TCAAACGTGAGAGTGTCTAACG	103
	Reverse: CCGGGCCGAAGAGATTTCTG	
*PCNA*	Forward: ACCTGCAGAGCATGGACTCG	83
	Reverse: GCAGCGGTATGTGTCGAAGC	
*KI67*	Forward: ATCATTGACCGCTCCTTTAGGT	226
	Reverse: GCTCGCCTTGATGGTTCCT	
*ITIH1*	Forward: CTCTCGCTTTGCCCACTACG	61
	Reverse: CCTGGCTTTATCAGCATTGTTGA	
*ITIH2*	Forward: ATCCCCATAAACGGAAACTCAGA	198
	Reverse: GGCCACCCGAGAAGTAATAGTG	
*ITIH3*	Forward: TGCTCACAATGTTGTCACCAC	214
	Reverse: CTTGACCAAACCGGCTGTC	
*ITIH4*	Forward: CCTCCGTTGCAGCACAATATC	126
	Reverse: GCGGCAAGGTAATAGTAGGAAC	
*HAS2*	Forward: TGTGAGAGGTTTCTATGTGTCCT	144
	Reverse: ACCGTACAGTCCAAATGAGAAGT	
*TNFAIP6*	Forward: GGGATTCAAGAACGGGATCTTT	129
	Reverse: TCAAATTCACATACGGCCTTGG	

### Western blot

The collected samples were homogenated in radioimmunoprecipitation assay lysis solution (ComWin Biotech) containing protease and phosphatase inhibitors (ComWin Biotech) and PMSF (Solarbio). Protein content was measured using BCA Protein Assay Kit (Servicebio). Thereafter, the protein bands were separated using polyacrylamide gel electrophoresis and then transferred to a polyvinylidene fluoride membrane. After band transfer, the membrane was blocked with 5% skim milk powder (Nestle) at room temperature and incubated at 4 °C overnight with the appropriate primary antibodies listed: 4E-BP1 (1:1000 dilution, catalog no.: 9452S; CST), phospho-4E-BP1 (1:1000 dilution, catalog no.: 9451S; CST), p70-S6K1 (1:1000 dilution, catalog no.: 9202S; CST), phospho-p70S6K1 (1:1000 dilution, catalog no.: 9205S; CST), mTOR (1:1000 dilution, catalog no.: 2983S; CST), phospho-mTOR (1:1000 dilution, catalog no.: 5536S; CST), and α-tubulin (1:1000 dilution; catalog no.: B1052; Biodragon-immunotech). Next, the membrane was rinsed with Tris-buffered saline with Tween-20 (Servicebio) and incubated with the secondary antibody (goat anti-rabbit immunoglobulin G, 1:4000 dilution; goat antimouse immunoglobulin G, 1:4000 dilution, Biodragon-immunotech) for 60 min at room temperature. Finally, the protein blots were visualized with ECL chemiluminescent reagent kit (Servicebio). Photographs were acquired by a Chemiluminescence imager (Image Quant LAS 4000 mini).

### Ovarian moisture determination

Ovarian moisture was measured using dry-wet method. The wet weight (WW) of the ovarian samples was determined using an analytical balance (Sartorius). The ovarian samples were then transferred to a thermostatic drying oven for 60 min with the temperature set at 100 °C. After drying, the dry weight of the ovaries was determined. Ovarian moisture (%) was calculated according to the formula: (WW-dry weight)/WW × 100%.

### Hormone determination

The contents of estradiol and progesterone in serum were measured using radioimmunoassay. Briefly, serums were obtained from the whole blood by centrifugation at 3000 rpm for 10 min and stored at −20 °C. The detection kits were purchased from the Bioengineering Institute. Detection was undertaken by the North Institute of Biological Technology.

### ATP assay

ATP was measured by luciferinluciferase method using ATP Assay Kit (Beyotime). To detect the changes of ATP content during iFFA, ovaries were collected on PD14 and PD15, respectively. To explore the effect of rapamycin injection on ATP content, ovaries were collected on PD15. According to the manufacturer’s instruction, the collected ovaries were homogenated with 100 μl ATP lysis buffer and centrifuged at 12,000 rpm for 5 min at 4 °C. Thereafter, the supernatant (50 μl) was added to the detection plate containing the ATP detection working solution (50 μl). The luminance (relative light unit) was measured by an enzyme marker microplate reader (PerkinElmer), and the standard curve was also generated to calculate ATP contents in supernatant. Finally, the protein content of supernatant was determined using BCA Protein Assay Kit to normalize ATP content. The ATP content was thus expressed as picomole/milligram protein.

### NAD^+^/NADH assay

The ratio of NAD^+^/NADH was measured using the NAD^+^/NADH Assay Kit with WST-8 (Beyotime). To detect the changes of NAD^+^/NADH, ovaries were collected on PD14 and PD15, respectively. To explore the effect of rapamycin intake on NAD^+^/NADH, ovaries were collected on PD15. Thereafter, the collected ovaries were homogenated with the precooled lysis buffer (200 μl). To measure the total amount of “NAD^+^ + NADH,” the alcohol dehydrogenase working solution (90 μl) and chromogenic solution (10 μl) were successively added to the ovarian lysates. To detect the amount of NADH, the ovarian lysates were heated to 60 °C for 10 min, followed by centrifugation. The supernatant (20 μl) was then incubated with 90 μl alcohol dehydrogenase working solution at 37 °C for 10 min. Finally, the reaction system was added with 10 μl chromogenic solution and incubated in dark at 37 °C for 30 min. The absorbance at 450 nm was measured by a microplate reader. The standard curves were produced simultaneously to calculate the contents of “NAD^+^ + NADH” and NADH content. NAD^+^/NADH was calculated according to the formula: (“NAD^+^ + NADH” − NADH)/NADH.

### Enzyme assay

The activity of PDH was determined using PDH Activity Assay Kit (Boxbio). Briefly, ovaries were collected on PD14 and PD15 and then homogenated with 150 μl PDH lysis buffer. After centrifugation (12,000 rpm, 10 min, 4 °C), 100 μl supernatant was sucked and mixed with 150 μl PDH working solution. Finally, the absorbance was measured at 605 nm by the microplate reader at 0 and 60 s, respectively. The activity of α-ketoglutarate dehydrogenase was determined using α-ketoglutarate dehydrogenase (α-KGDH) Activity Assay Kit (Boxbio). According to the manufacturer’s instruction, the collected ovaries were homogenated with 150 μl α-KGDH lysis buffer. After centrifugation (12,000 rpm, 10 min, 4 °C), 100 μl supernatant was sucked and mixed with α-KGDH working solution (100 μl) and reagent VIII (50 μl). Finally, the absorbance was measured at 340 nm by the microplate reader at 0 and 120 s, respectively. Enzymatic activities were converted from the respective absorbance and normalized by the total protein amount.

### Statistics analysis

Statistical analyses were using GraphPad Prism 8.0 (GraphPad). Data were expressed as the mean ± SD. Two-tailed unpaired Student’s *t* test and one-way analysis of variance followed by Tukey’s post hoc test were used to analyze the statistical significance between two groups and among multiple groups, respectively. Chi-squared test was used in the comparison between the percentages. The statistical significance was set at *p* value < 0.05.

## Data availability

The RNA-Seq data reported in this study have been deposited in the Gene Expression Omnibus under accession number GSE207958 (https://www.ncbi.nlm.nih.gov/geo/query/acc.cgi?acc=GSE207958; Secure token: alexccgunbwnbgx). Any information required to reanalyze the data reported in this article is available upon reasonable request.

## Supporting information

This article contains supporting information.

## Conflict of interest

The authors declare that they have no conflicts of interest with the contents of this article.
