# Supplementary material for: The FSH–mTOR–CNP signaling axis initiates follicular antrum formation by regulating tight junction, ion pumps, and aquaporins
Source: J Biol Chem. 2023 Jul 5;299(8):105015. doi: 10.1016/j.jbc.2023.105015 (PMC10424218; doi:10.1016/j.jbc.2023.105015)
Supplement: Supporting Figures S1–S17 [file mmc1.docx]

**Supporting information**

**The *FSH-mTOR-CNP* signaling axis initiates follicular antrum formation by regulating tight junction, ion pumps, and aquaporins**

Xiaodong Wang^1Ϯ^, Shanshan Zhou^1Ϯ^, Zian Wu^1^, Ruiyan Liu^1^, Zaohong Ran^1^, Jianning Liao^1^, Hongru Shi^1^, Feng Wang^2^, Jianguo Chen^1^, Guoshi Liu^3^, Aixin Liang^1^, Liguo Yang^1^, Shujun Zhang^1^, Xiang Li^1^, Changjiu He^1*^

^Ϯ^ These authors contributed equally to this work.

*Corresponding Author: Changjiu He, email: [chungjoe@mail.hzau.edu.cn](mailto:chungjoe@mail.hzau.edu.cn)

**This supporting information file contains:**

Figures S1 – S17

**
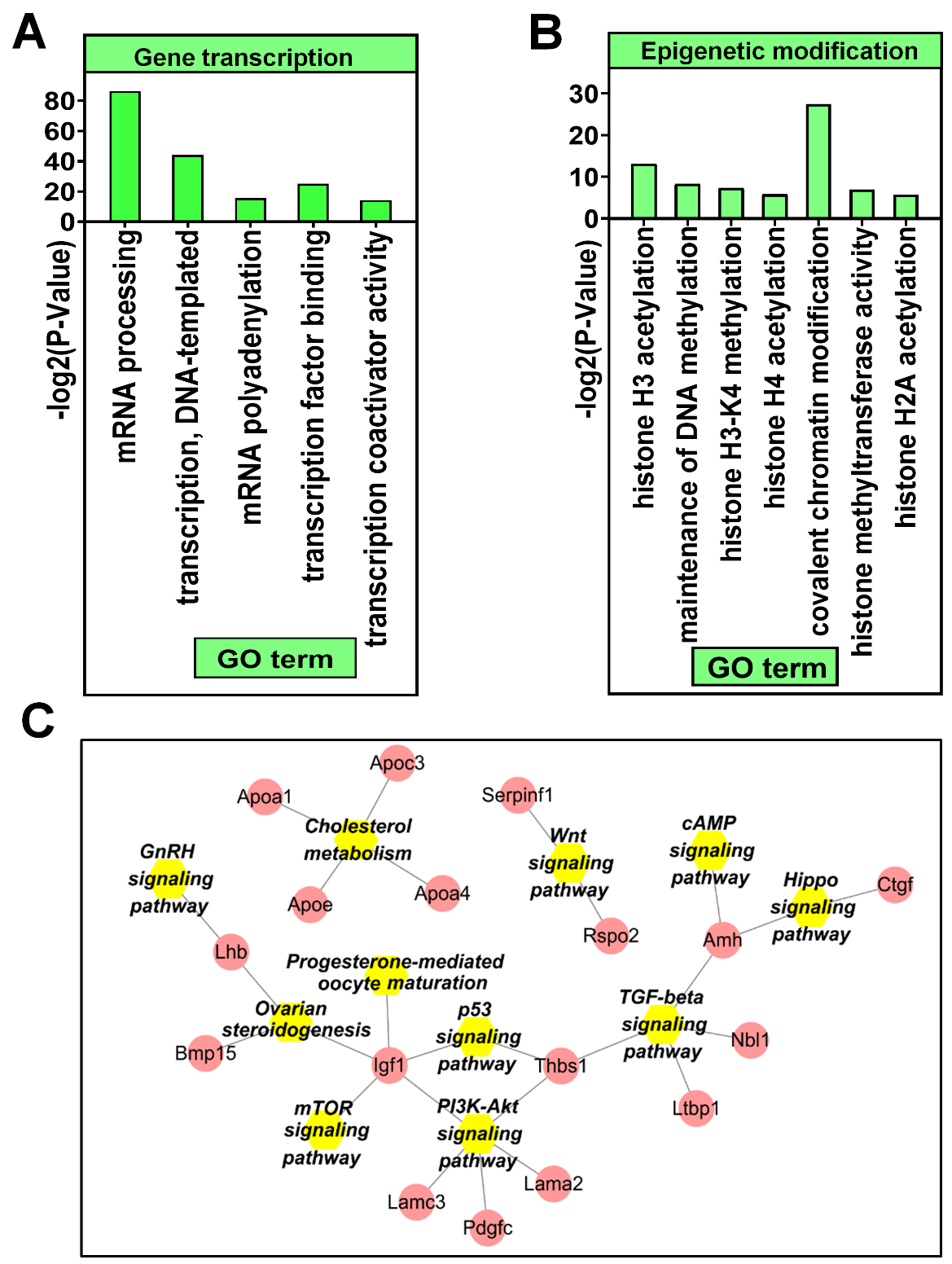
**

**Figure S1.** (A) Cytological events related to “Transcription” were identified by GO analysis. (B) Cytological events related to “Epigenetic modification” were identified by GO analysis. (C) KEGG analysis of the pathways to which upregulated secretory proteins belong (related to Figure 2).


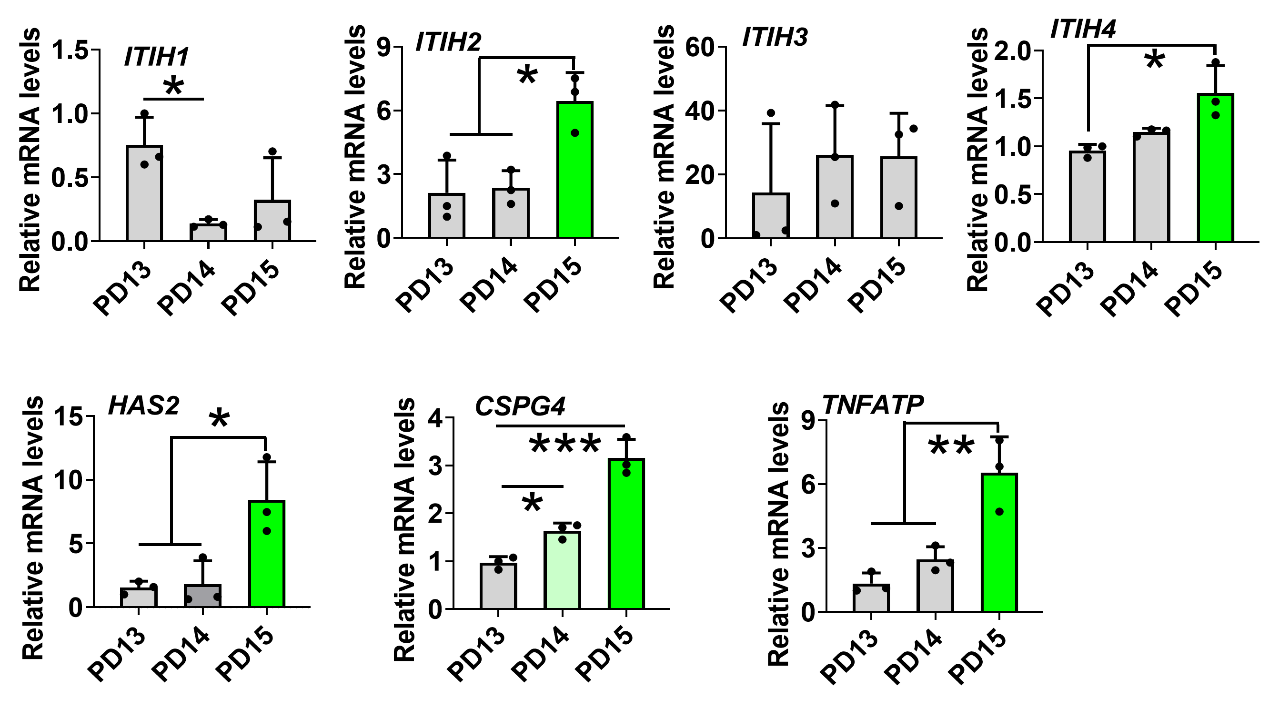


**Figure S2. Changes in expression of genes encoding large macromolecule hydrophilic secretory proteins during iFFA (related to Figure 2).** n = 3 biologically independent ovaries. Statistical signiﬁcance were determined using one-way ANOVA followed by Tukey’s post hoc test, values were mean ± SD. *P<0.05, **P<0.01, ***P<0.001. The experiments were repeated two times independently, and similar results were obtained.

**
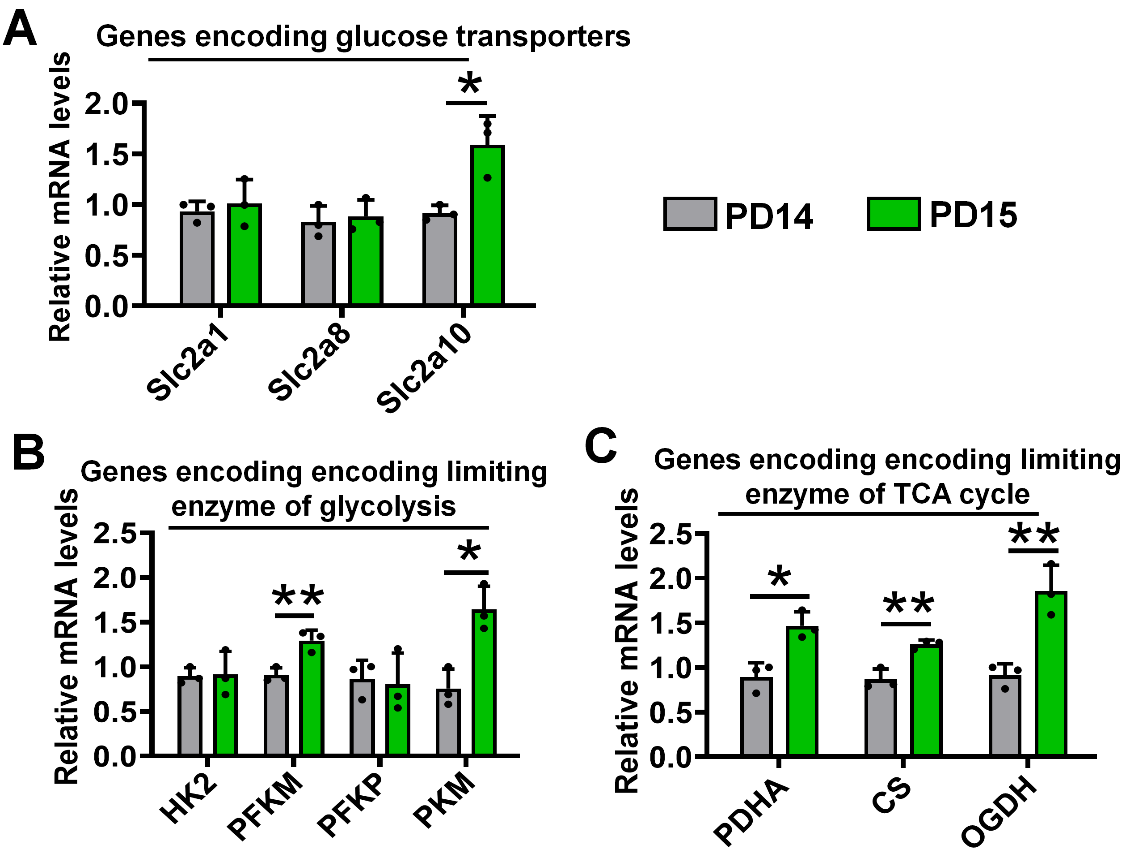
**

**Figure S3. Changes in expression of genes during iFFA (related to Figure 2).** (A) Genes encoding glucose transporters**.** (B) Genes encoding limiting enzyme of glycolysis**.** (C) Genes encoding rate-limiting enzymes of tricarboxylic acid (TCA) cycle**.** n = 3 biologically independent ovaries. Statistical signiﬁcance was determined using two-tailed unpaired Student’s t-test, values were mean ± SD. *P<0.05, **P<0.01,. The experiments were repeated three times independently, and similar results were obtained.


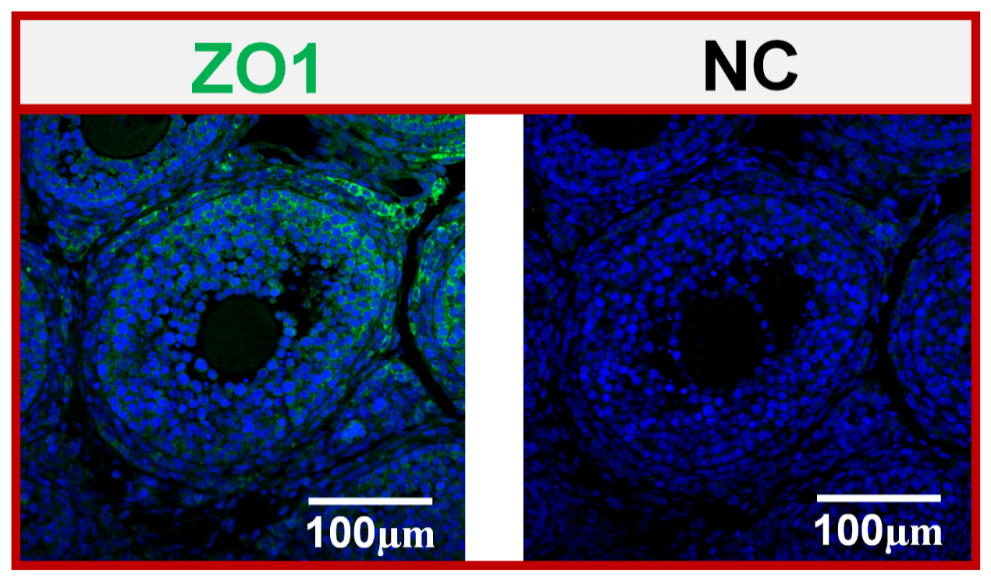


**Figure S4. Immunofluorescence showed that tight junction formed in the large preantral follicle (related to Figure 3).** ZO1 was used as a marker protein to recognize tight junction. NC: negative control.


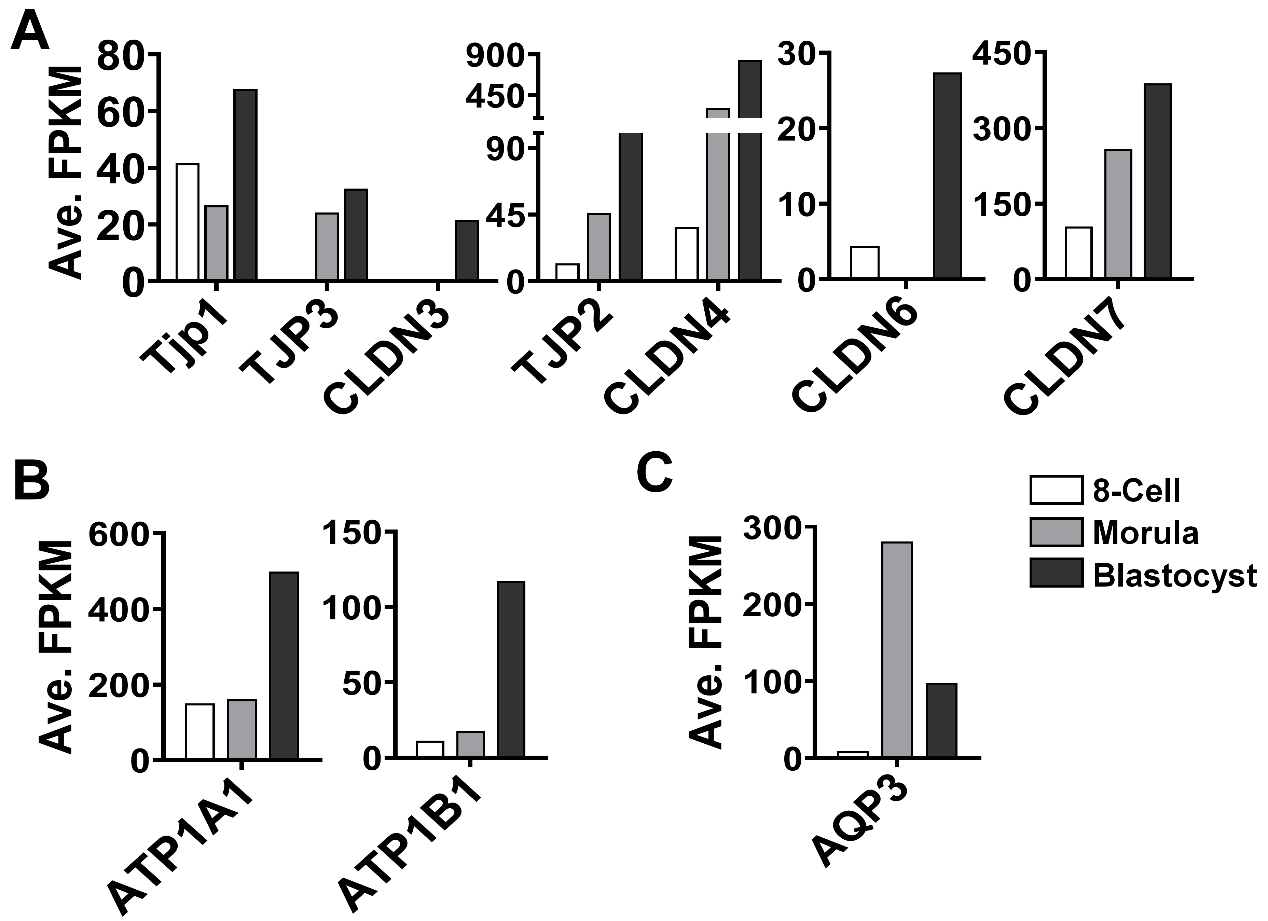


**Figure S5.** **Transcriptome showed that genes encoding tight junction, ion pumps, and aquaporins were up-regulated during blastocoel formation (related to Figure 3).** (A) Changes in expression of genes encoding the core components of tight junction during blastocoel formation. (B) Changes in expression of genes encoding ion pumps during blastocoel formation. (C) Change in expression of *AQP3* during blastocoel formation.

**
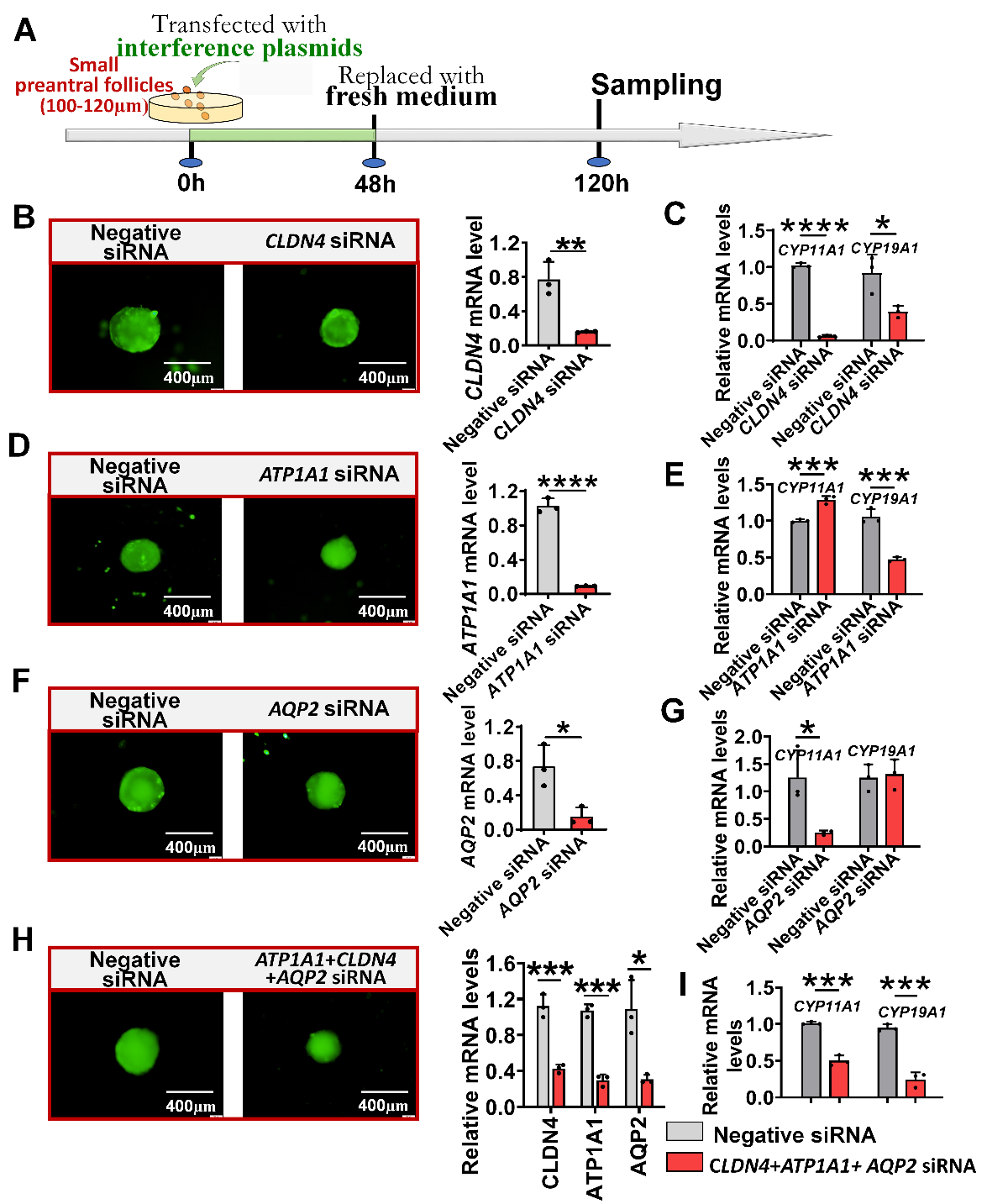
**

**Figure S6.** **RNA interference was performed on the follicles and its effect on genes associated with steroidogenesis was validated (related to Figure 3).** (A) Experimental design of RNA interference. (B) The efficiency of *CLDN4* interference. Green fluorescence indicated successful transcription of interfering plasmids in follicles. (C) Changes in the expression of *CYP11A1* and *CYP19A1* after *CLDN4* knockdown, n = 3 follicular samples. (D) The efficiency of *ATP1A1* interference. (E) Changes in the expression of *CYP19A1* after *ATP1A1* knockdown, n = 3 follicular samples. (F) The efficiency of *AQP2* interference. (G) Changes in the expression of *CYP11A1* after *AQP2* knockdown, n = 3 follicular samples. (H) The efficiency of *CLDN4+ATP1A1+AQP2* interference. (I) Changes in the expression of *CYP11A1* and *CYP19A1* after *CLDN4+ATP1A1+AQP2* knockdown, n = 3 follicular samples. Statistical signiﬁcance was determined using two-tailed unpaired Student’s t-test, values were mean ± SD. *P<0.05, **P<0.01, ***P<0.001, ****P<0.001. The experiments were repeated two times, and similar results were obtained.


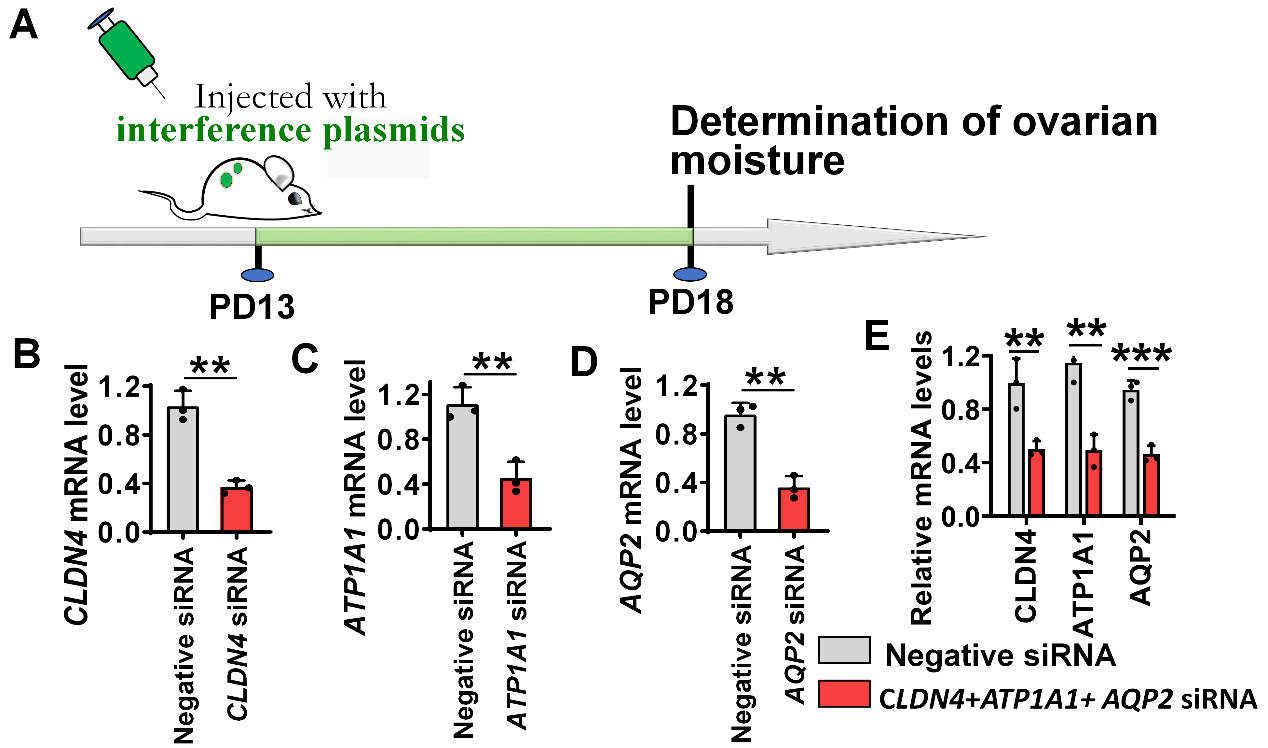


**Figure S7.** **Validation of the efficiency of RNA interference in the ovary (related to Figure 3).** (A) Experimental design of RNA interference. (B) The efficiency of *CLDN4* interference (C) The efficiency of *ATP1A1* interference. (D) The efficiency of *AQP2* interference. (E) The efficiency of *CLDN4+ATP1A1+AQP2* interference. n = 3 biologically independent ovaries. Statistical signiﬁcance was determined using two-tailed unpaired Student’s t-test, values were mean ± SD. **P<0.01, ***P<0.001. The experiments were repeated two times and similar results were obtained.


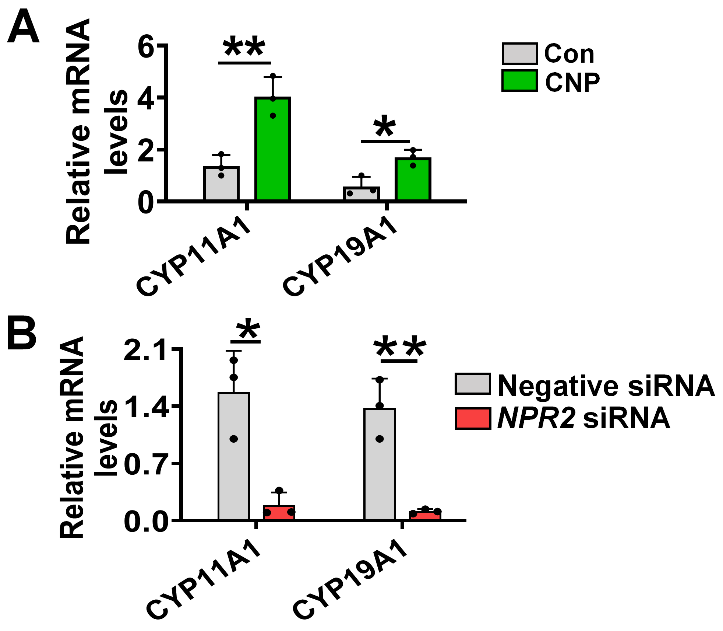


**Figure S8.** **CNP regulated the expression of genes controlling** **steroidogenesis (related to Figure 4).** (A) Changes in the expression of *CYP11A1* and *CYP19A1* after CNP injection. n = 3 biologically independent ovaries. (B) Changes in the expression of *CYP11A1* and *CYP19A1* after *NPR2* knockdown, n = 3 follicular samples. Statistical signiﬁcance was determined using two-tailed unpaired Student’s t-test, values were mean ± SD. *P<0.05, **P<0.01.


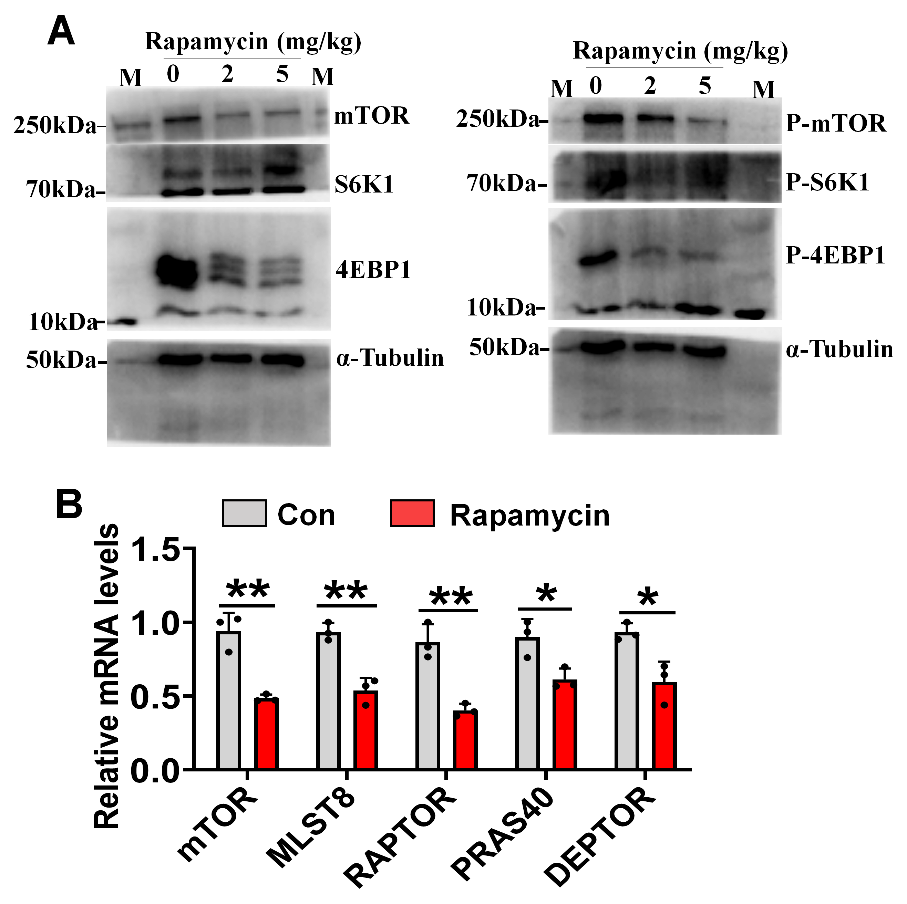


**Figure S9. Determination of the optimal dose of rapamycin to inhibit the mTOR pathway (related to Figure 5)**. (A) Effect of rapamycin injection on the activity of mTOR pathway. M: marker. (B) Changes in expression of genes in mTOR pathway after Rapamycin injection, n = 3 biologically independent ovaries. Statistical signiﬁcance were determined using two-tailed unpaired Student’s t-test, values were mean ± SD. *P<0.05, **P<0.01. The experiments were repeated three times independently, and similar results were obtained.


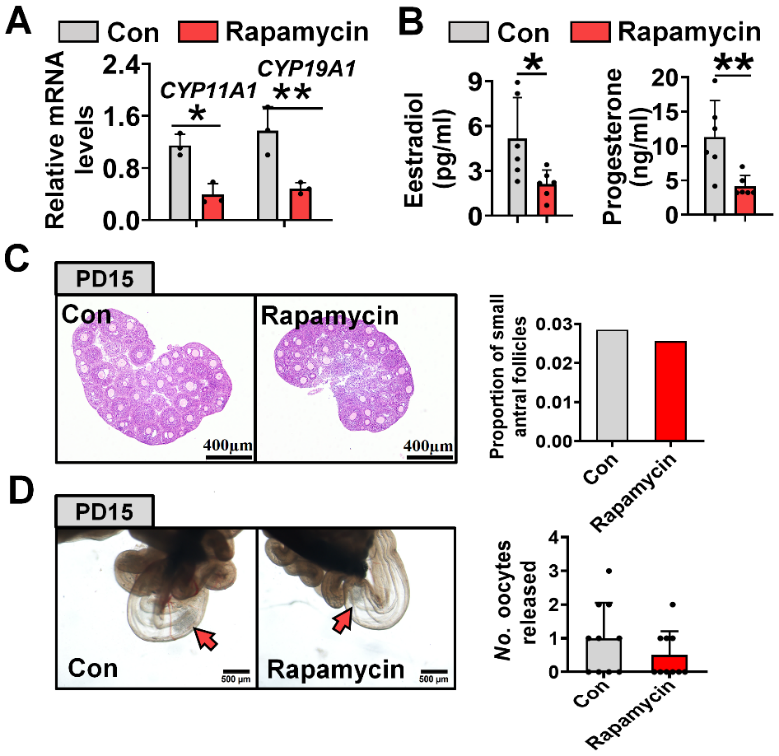


**Figure S10.** **Effects of rapamycin injection on the proportion of small antral follicles in the ovary of PD15 (related to Figure 5).** (A) Effect of rapamycin injection on the expression of *CYP11A1* and *CYP19A1*, n = 3 biologically independent ovaries. (B) Effect of rapamycin injection on estradiol and progesterone, n = 6 mice. (C) Effect of rapamycin injection on the proportion of small antral follicles, n = 3 biologically independent ovaries. (D) Superovulation was performed to verify the effect of rapamycin on the proportion of small antral follicles, n = 10 oviducts, collected from 5 mice. The red arrow indicates the oocytes released into the oviduct. Statistical signiﬁcance was determined using *Chi*-square test (Fig. S10C), and two-tailed unpaired Student’s t-test, values were mean ± SD. *P<0.05, **P<0.01. The experiments were repeated three times independently, and similar results were obtained.

**
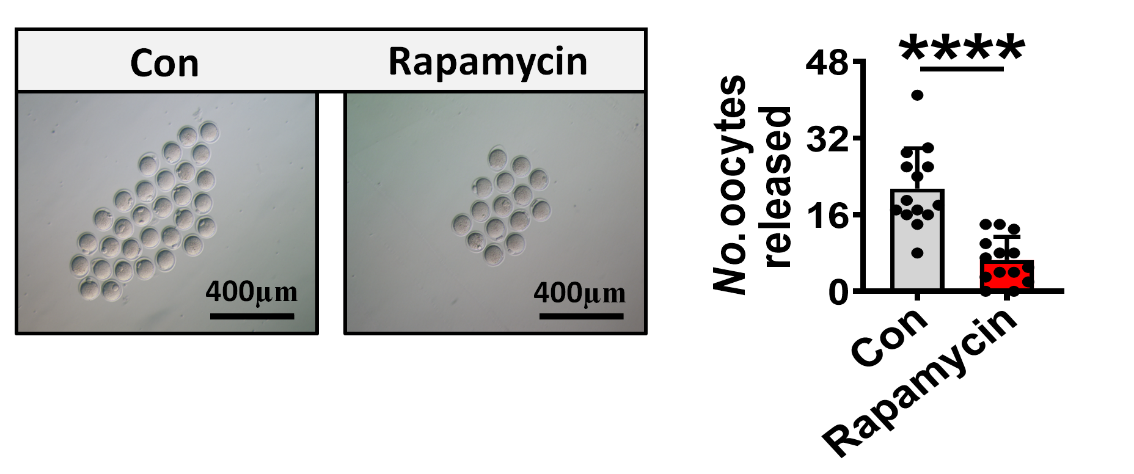
**

**Figure S11. Superovulation was performed to verify the effect of rapamycin injection on the proportion of small antral follicles (related to Figure 5).** n = 14 oviducts, collected from 7 mice. Statistical signiﬁcance was determined using two-tailed unpaired Student’s t-test, values were mean ± SD. ****P<0.0001.The experiment was repeated three times independently, and similar results were obtained.


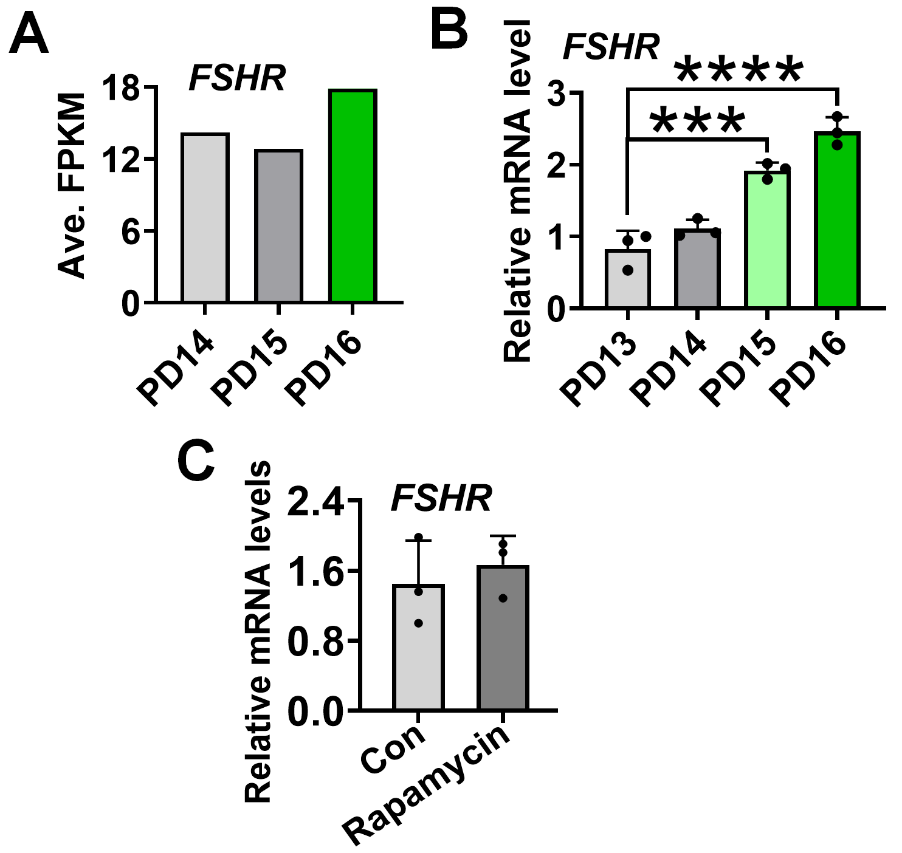


**Figure S12. *FSHR* was upregulated during iFFA and was not affected by rapamycin tratment (related to Figure 6)**. (A) Transcriptome showed that the FPKM value of *FSHR* increased during iFFA. (B) qRT-PCR suggested that the expression of *FSHR* was upregulated during iFFA, n = 3 biologically independent ovaries. (C) Effect of rapamycin injection on the expression of *FSHR* in ovary, n = 3 biologically independent ovaries. Statistical signiﬁcance was determined using one-way ANOVA followed by Tukey’s post hoc test (B), and two-tailed unpaired Student’s t-test (C). Values were mean ± SD. ***P<0.001, ****P<0.0001. B, C was repeated three times independently, and similar results were obtained.


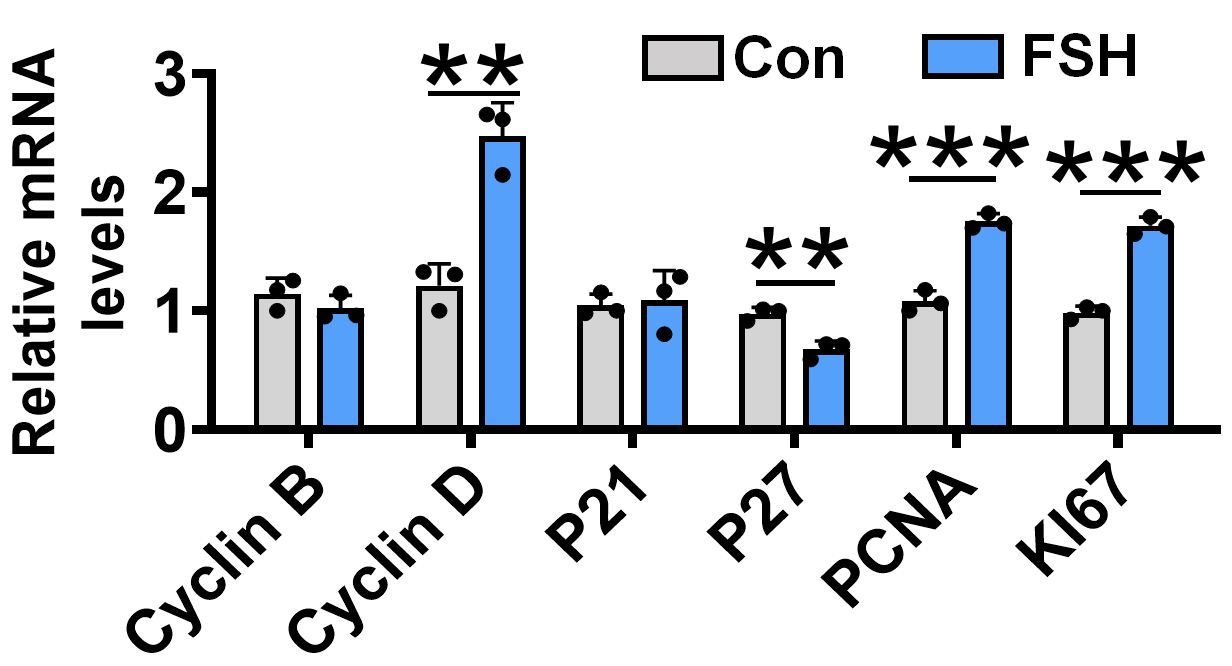


**Figure S13. FSH-injection upregulated the expression of proliferation-related genes (related to Figure 6)**. n = 3 biologically independent ovaries. Statistical signiﬁcance was determined using two-tailed unpaired Student’s t-test, values were mean ± SD. **P<0.01, ***P<0.001. The experiments were repeated three times independently, and similar results were obtained.


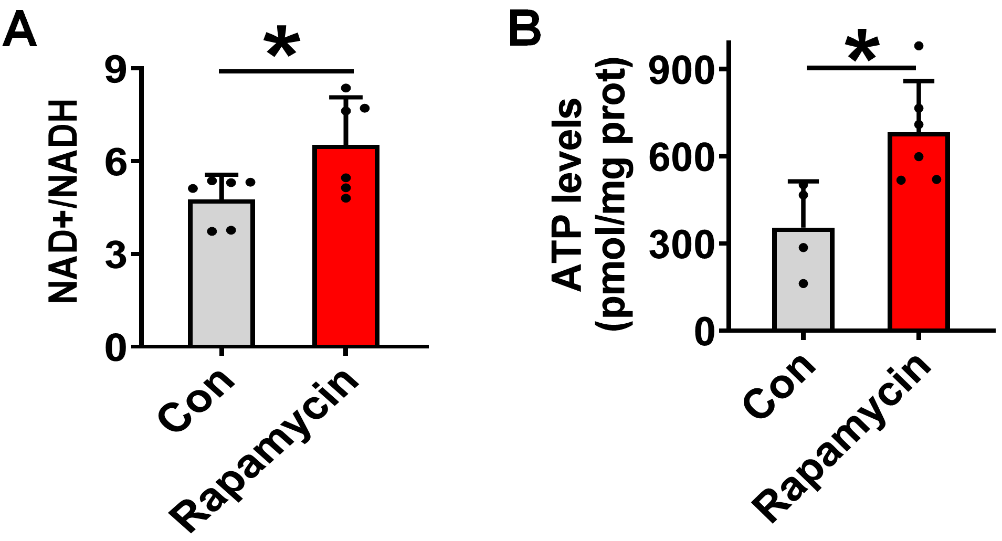


**Figure S14. The effect of rapamycin-injection on energy production in ovary of PD15 (related to Figure 5)**. (A) The ratio of NAD^+^/NADH in ovaries increased after Rapa injection, n = 6. (B) ATP contents in ovaries increased after rapamycin injection, n = 4 (Con), 6 (Rapamycin) biologically independent ovaries. Statistical signiﬁcance was determined using two-tailed unpaired Student’s t-test, values were mean ± SD. *P<0.05. The experiments were repeated three times independently, and similar results were obtained.


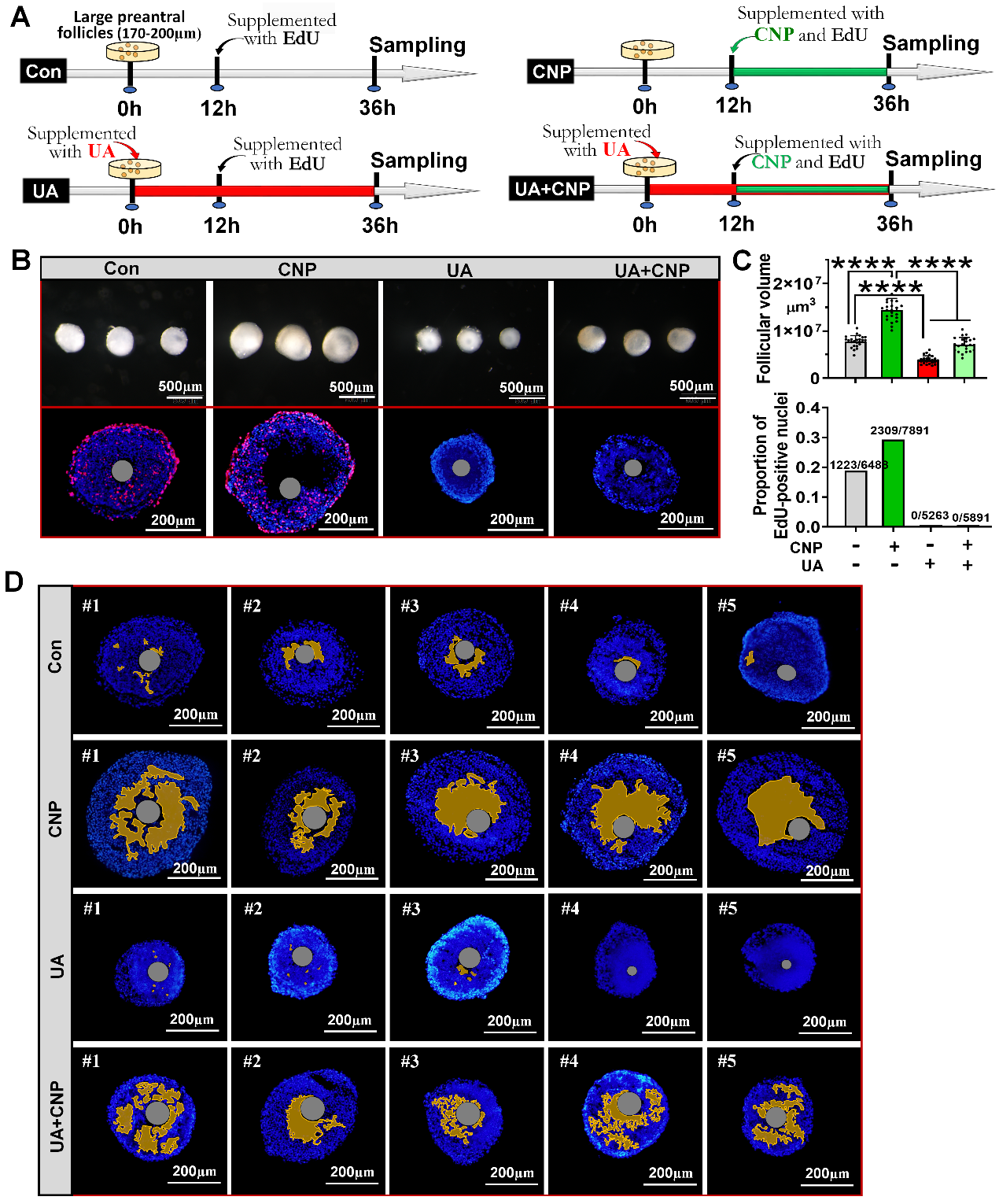


**FIGURE S15.** **CNP induced iFFA when follicular cell proliferation was blocke**d **(related to Figure 4)**. (A) Experimental design. UA is short for Urolithin A. (B) Representative photographs of follicles and follicular slices in each group, the red dots represented EdU-positive nuclei. (C) Changes in the follicular volume (n = 23-24 follicles) and the proportion of EdU-positive nuclei (n = 6 slices from independent follicles), (D) Changes in follicular antrum, n = 6 slices from independent follicles. The areas covered in yellow were follicular antra, and the areas covered in gray were the oocytes. Statistical signiﬁcance was determined using one-way ANOVA followed by Tukey’s post hoc test, values were mean ± SD. ****P<0.0001.


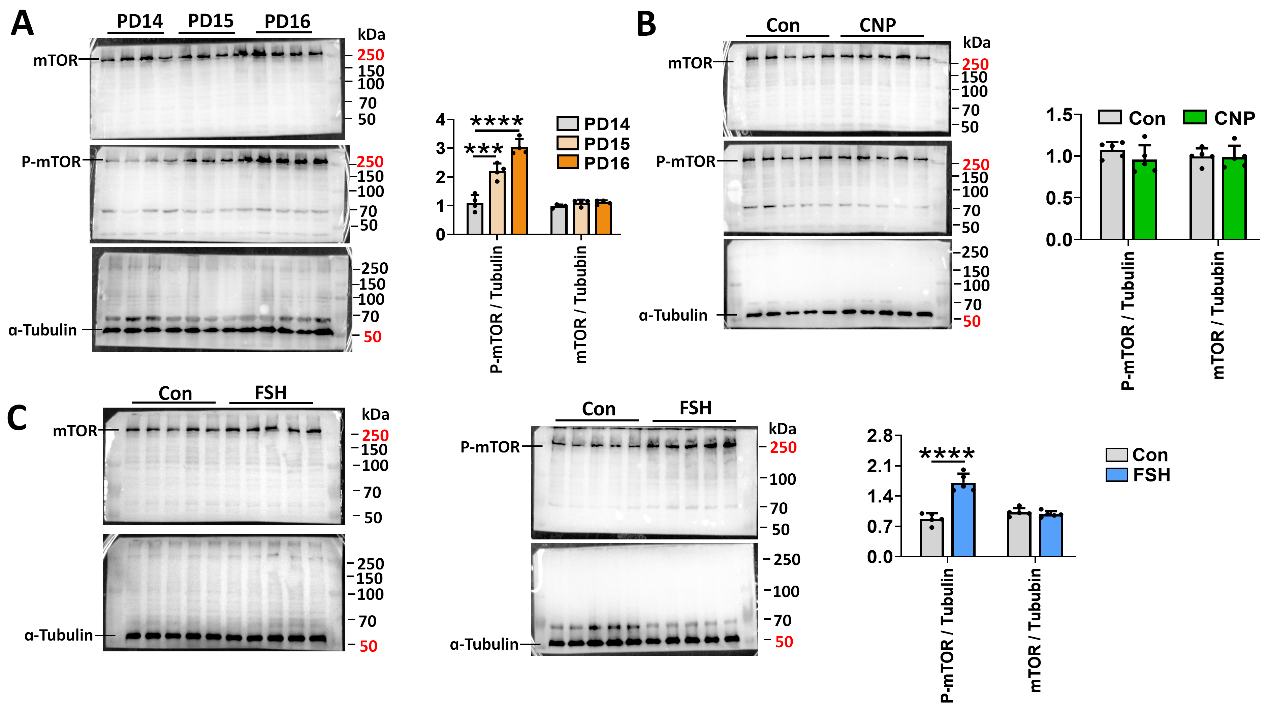


**FIGURE S16**. **The full western blots.** (A) Changes in the phosphorylation of mTOR in ovaries during iFFA (related to Figure 2N). (B) Effect of CNP injection on mTOR phosphorylation. (C) Effect of FSH injection on mTOR phosphorylation (related to Figure 6E). Statistical signiﬁcance was determined using one-way ANOVA followed by Tukey’s post hoc test (A), and two-tailed unpaired Student’s t-test (B, C). Values were mean ± SD. ***P<0.001, ****P<0.0001.

**
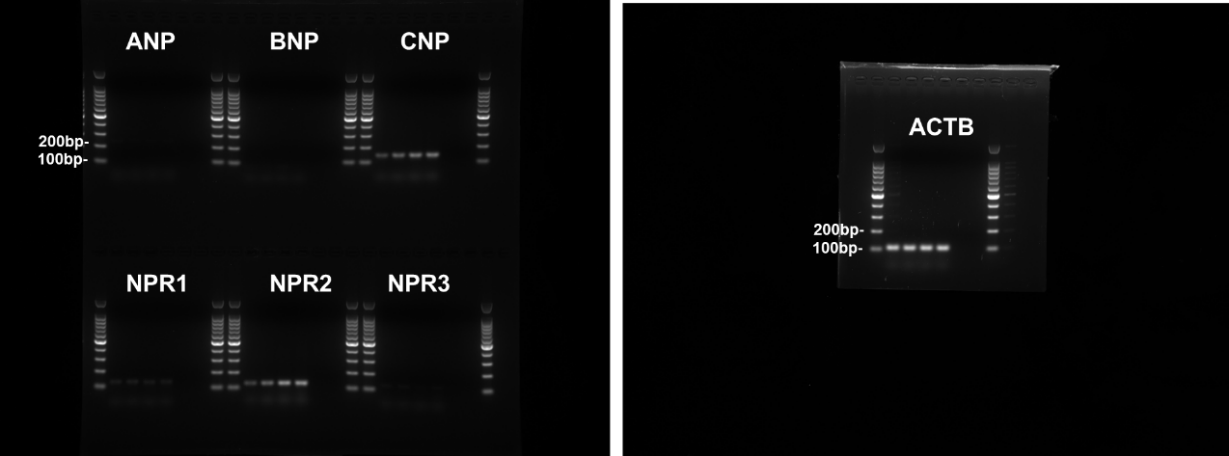
**

**Figure S17. Original agarose gel eletrophoresis in Figure 4A**
